# Co-benefits of designing communities for active living: an exploration of literature

**DOI:** 10.1186/s12966-015-0188-2

**Published:** 2015-02-28

**Authors:** James F Sallis, Chad Spoon, Nick Cavill, Jessa K Engelberg, Klaus Gebel, Mike Parker, Christina M Thornton, Debbie Lou, Amanda L Wilson, Carmen L Cutter, Ding Ding

**Affiliations:** Active Living Research, University of California, San Diego, USA; Department of Family and Preventive Medicine, 3900 5th Avenue, Suite 310, San Diego, CA 92119 USA; Cavill Associates Ltd, 185A Moss Lane, Bramhall, Stockport, Cheshire SK7 1BA UK; University of California San Diego/San Diego State University, Public Health Joint Doctoral Program, 3900 5th Avenue, Suite 310, San Diego, CA 92119 USA; James Cook University, Centre for Chronic Disease Prevention, PO Box 6811, Cairns, QLD 4870 Australia; Progress Health Partnerships Ltd, 5 Elmfield Road, Wigan, WN1 5RG UK; The University of Sydney, Sydney School of Public Health, Edward Ford Building (A27), Sydney, NSW 2006 Australia

**Keywords:** Healthy communities, Physical activity, Built environment, Parks, Trails, Land use, Urban design, Schools, Workplace, Transportation

## Abstract

To reverse the global epidemic of physical inactivity that is responsible for more than 5 million deaths per year, many groups recommend creating “activity-friendly environments.” Such environments may have other benefits, beyond facilitating physical activity, but these potential co-benefits have not been well described. The purpose of the present paper is to explore a wide range of literature and conduct an initial summary of evidence on co-benefits of activity-friendly environments. An extensive but non-systematic review of scientific and “gray” literature was conducted. Five physical activity settings were defined: parks/open space/trails, urban design, transportation, schools, and workplaces/buildings. Several evidence-based activity-friendly features were identified for each setting. Six potential outcomes/co-benefits were searched: physical health, mental health, social benefits, safety/injury prevention, environmental sustainability, and economics. A total of 418 higher-quality findings were summarized. The overall summary indicated 22 of 30 setting by outcome combinations showed “strong” evidence of co-benefits. Each setting had strong evidence of at least three co-benefits, with only one occurrence of a net negative effect. All settings showed the potential to contribute to environmental sustainability and economic benefits. Specific environmental features with the strongest evidence of multiple co-benefits were park proximity, mixed land use, trees/greenery, accessibility and street connectivity, building design, and workplace physical activity policies/programs. The exploration revealed substantial evidence that designing community environments that make physical activity attractive and convenient is likely to produce additional important benefits. The extent of the evidence justifies systematic reviews and additional research to fill gaps.

**Electronic supplementary material:**

The online version of this article (doi:10.1186/s12966-015-0188-2) contains supplementary material, which is available to authorized users.

## Introduction

Physical inactivity accounts for 5 million deaths annually worldwide [1]. Most people are not sufficiently active, and physical activity is declining in many countries [2]. This is a global problem with the biggest burden in low and middle income countries [3]. Increasing physical activity is a priority of the United Nations through its non-communicable disease initiative [4].

Physical activity has been engineered out of people’s lives through urban planning and transportation investments that favor travel by automobile, labor-saving devices at home and in the work place, and a proliferation of electronic entertainment options [5,6]. Built environments are worthy of special attention because they can affect virtually all residents of a community for many decades. The United Nations [4], World Health Organization [7], national physical activity plans [8], U.S. Guide to Community Preventive Services [9,10], U.S. Institute of Medicine [11], and other scientific groups worldwide [12,13] have identified creating built environments and implementing policies that support active living as essential for increasing physical activity and improving health.

Many decisions affecting physical activity environments occur at the local government level. Though mayors, city council members, and other officials work every day to balance competing interests, they likely do not consider that environments supporting physical activity could produce additional benefits for their communities. For example, changing zoning codes to favor mixed use developments can enhance property values and reduce carbon emissions [14,15]. Having parks in neighborhoods has been linked with physical and mental health benefits [16].

There is no resource that examines the wide range of potential co-benefits of communities designed to support active living, which can be called “activity-friendly environments”. Therefore, the aim of the current study was to compile evidence about the relation of activity-friendly environmental attributes to multiple potential outcomes. The expectation was that several co-benefits would be documented, but negative effects were also included. The intent of the present exploration of the literature was to provide an initial summary of the evidence on co-benefits that may be useful for determining whether and which systematic reviews are justified, identifying areas for further research, and educating policy makers about likely co-benefits.

## Methods

The present literature review covered diverse topics across multiple academic and practice fields, so dozens of systematic reviews were not feasible. Therefore an exploratory approach was taken to provide an initial overview of the potential co-benefits of communities designed for active living. We searched both scientific and gray literature. Gray literature was included because we expected many of the topic areas to be rarely addressed in the scientific literature but studied by government agencies and policy groups. The objective of the literature exploration was not to systematically review or quantify all evidence, but to create a profile of the potential multiple benefits, and negative effects, of each environmental feature as a tool for policy-making and a guide for future research.

### Search areas

#### Built environment attributes

Specific built and social environment attributes in five settings (open spaces/parks/trails, urban design/land use, transportation, schools, workplaces/buildings) that research had shown to be related to physical activity were identified and used to structure the search (Table 1). The environmental attributes represented a multi-level conceptualization, based on ecological principles of multiple levels of influence on behavior and interactions across levels [17]. Proximity of activity-promoting settings was the most basic characteristic that could affect physical activity, such as proximity of parks or shops to homes. A second consideration was the quality or design of the setting, such as physical activity facilities within parks or quality of sidewalks. The third consideration was that social environments could interact with built environment features in affecting outcomes. For example, events and programs could improve use of well-designed parks, and social disorder like graffiti and boarded-up buildings could negate the benefits of well-designed streetscapes.

**Table 1:** **Built and social environment features with evidence of association with physical activity**

**Setting**	**Feature**	**Description**	**Reference**
Open Spaces/Parks/Trails	Design features	Size, amenities, physical activity facilities	[18-21]
	Presence/proximity	Existence of and distance to	[6,18,22]
	Trails	Proximity to and design of	[6,20]
	Programs, promotion, and events	Park-based programming	[19]
	Park incivilities/civilities	Existence or lack of graffiti, litter, anti-social behavior (public drinking, loitering)	[20,23,24]
	Public gardens	Presence	[19]
Urban Design/Land Use	Density	Population and housing density	[6,18,22]
	Mixed land use	Mix of destinations, distance to destinations	[6,18,22]
	Streetscale pedestrian design	Including buffers between street and sidewalk, building set-back from sidewalk, form based codes, street lights, etc.	[10,25]
	Greenery	Street trees/shrubbery, gardens	[18,22]
	Incivilities	Graffiti, vacant/dilapidated buildings, litter, anti-social behavior (public drinking, loitering)	[18,23,24]
	Accessibility & street connectivity	Density of intersections in street network	[18,20,26]
Transportation	Pedestrian/bicycle infrastructure	Sidewalks, bike lanes/paths, bike parking	[6,18,22,25]
	Crosswalk markings	Crosswalk and intersection quality	[25,27]
	Traffic calming	Speed bumps, curb-cuts, road diet, other engineering infrastructure	[27,28]
	Public transportation	Proximity to or density of bus, train stops	[6,20,22]
	Traffic speed/volume		[18]
	Safe routes to school	Engineering, programming, promotion and events	[6,21]
	Ciclovia/play streets	Opening streets for walking, bicycling, rolling, play	[26,29]
	Managed parking	Restricted parking access	[30]
Schools	School siting	Location of school, distance from residences (suburban, urban, rural)	[31]
	Recreation facilities	Physical education (PE) facilities and equipment, presence of PE teachers	[32,33]
	Shared use agreements	Community use of school facilities for physical activity	[33,34]
Buildings/Workplaces	Building siting	Distance to residences, accessibility by public transit	[35]
	Mixed land use around worksite	Mix of destinations, distance to destinations	[6,18,22]
	Building site design	Design of property that building sits upon with physical activity options	[35]
	Building design	Stair design, exercise equipment presence, shower/locker presence, skip-stop elevators	[35,36]
	Worksite physical activity policies and programs	Exercise classes, discounted gym membership, active transportation promotion policies, parking cash out programs, point-of-decision prompts	[37,38]
	Workplace furniture design	Sit-stand desks	[39]

#### Co-benefits/outcomes

Based on the input of authors and informants, the following co-benefit outcomes were included in the searches: physical health, mental health, safety/injury prevention, social benefits, economic benefits, and environmental sustainability focusing on carbon emissions and air pollution (Table 2). These outcomes were defined as “co-benefits” because they were expected benefits of activity-friendly environments in addition to increased physical activity.

**Table 2:** **Outcomes of activity-supportive built and social environments examined in searches**

**Outcome/co-benefit**	**Description**
Physical health	Chronic diseases, obesity
Mental health	Depression, anxiety, well being, quality of life
Social benefits	Neighborhood/social cohesion, human capital
Environmental sustainability benefits	Carbon dioxide emissions, pollutants
Safety/Injury prevention	Crime, violence, injury, pedestrian/bicycle and car crashes
Economic benefits	Land value, governmental infrastructure costs, real estate profitability, productivity/job performance, health care costs, economic performance of cities

### Search strategies

#### Snowball sampling to identify sources

Through the Active Living Research (ALR, www.activelivingresearch.org) network, 20 experts in various disciplines were contacted to nominate 1) groups/organizations working on the built environment and non-physical activity co-benefits, 2) key reports and papers, both peer-reviewed and gray literature, 3) websites, 4) case studies of cities that have implemented activity-friendly built environment changes, 5) recommendations for other experts. Of the 20 experts contacted, 13 provided input.

#### Supplementary literature search

Authors conducted additional literature searches on environmental features and each co-benefit outcome to supplement the expert input. Literature searches were conducted November 2013 through February 2014 using combined search terms of environment features “and” co-benefit outcomes. Abstractors were instructed to use multiple synonyms for search terms because terms vary by discipline.

Search engines included Scopus, PubMed, Google Scholar, ISI Web of Science, MEDLINE, PsychINFO, Academic Search Premier, ClimateArk, and Google. Searches specific to European studies and carbon emission outcomes were conducted by invited international experts to enhance coverage of these topics. Due to the breadth of the overall search and differences across topics, reviewers developed search protocols specific to the topic area, but some guidelines were provided. Abstractors were encouraged to search for specialized search engines in their assigned fields. The initial goal was to be inclusive in finding relevant sources of information. For scientific literature, reviewers were instructed to find systematic or non-systematic reviews first. If reviews were located, then the individual studies did not need to be searched, except for publications since the latest review. In cases where a review paper did not provide adequate specificity or quantification in the findings, selected primary studies from that review paper were abstracted to illustrate specific findings. If reviews were not located, then individual studies were searched. For gray literature, reports from credible organizations were targeted, from such groups as government agencies, academic centers, and selected advocacy groups. Newspapers, magazines, and blogs were not searched, except to identify citations of or links to more credible reports.

### Data extraction

During the data extraction process, basic information on the built environment feature, co-benefit, study sample characteristics, study methods, and major findings were coded in tables specific to each of the five settings. Then the strength of each piece of evidence was graded based on the source, and the direction of each association was noted (Table 3). To simplify interpretation, “ + ” denotes that a physical activity-promoting environmental feature was associated with a co-benefit in a “favorable” direction. For example, having parks nearby was associated with better mental health or fewer carbon emissions. Similarly, “-” denoted that a physical activity-promoting feature of the environment was inversely associated with a co-benefit. For example, higher residential density was associated with more air pollution. A code “0” represented lack of significant association in either direction or inconsistent findings. Due to the number and diverse types of studies from different fields, it was not possible to grade the quality of each study, as is done in systematic reviews. Extraction tables were cross-checked by other staff for accuracy and clarity.

**Table 3:** **Scoring methods for summarizing the evidence**

**Score**	**Type of evidence**
4.5	Peer-reviewed, systematic review paper (including meta-analysis)
4	Peer-reviewed, non-systematic review paper (from scientific literature) or non-peer-reviewed review paper (from gray literature)
3.5	Any (singular) peer-reviewed study
3	Any (singular) non peer-reviewed study, such as a technical report from a government agency or academic center
2	Non-analytic studies (for example, case reports, case series, simulations) or advocacy report without a clear literature review
1	Expert opinion, formal consensus
**Score**	**Direction of association**
+	A favorable association was found between feature and co-benefit (feature was associated with “better” level of co-benefit
-	An unfavorable association was found between feature and co-benefit (feature was associated with “worse” level of co-benefit
0 (zero)	No association or inconsistent evidence was found between feature and co-benefit

### Synthesizing the findings

To illustrate areas with strong evidence as well as research gaps, a matrix was created for each of the five settings that summarized evidence of associations between built environment features and co-benefits. Using a quasi-quantitative approach, results were summarized by summing the weighted evidence from each resource. Briefly, each piece of evidence was scored based on the source and type of study/report (i.e., “weights”) and the direction of association. The weighted scores for associations in each direction were summed for each direction of effect category (“ + ”, “-”, “0”), and recorded in the online tables. Thus, there was a weighted score for each direction, such as 24 “ + ”, 7 “-”, and 4 “0”. “Net” scores were calculated by subtracting the weighted negative and zero scores from the weighted positive scores. In the preceding example, 24 minus (7 + 4) equals 13 (summary score). To be conservative, negative and zero findings were subtracted from positive findings, so the summary scores roughly indicated both the quantity and quality of the evidence. To make the summarization process even more conservative, only resources with quality scores of 3 or above were included, as resources with a lower score, such as unreferenced advocacy documents and consensus reports, lacked credibility.

Cells in each summary table were labeled based on summary scores. Table 4 presents the summary labels. A net score of 15 or above was considered strong evidence ([+++] or [−−-]), as this was equivalent to more than three systematic reviews consistently supporting the association between an environmental attribute and a co-benefit. Scores of 10–14 ([++] or [−−]), and 4–9 ([+] or [−]), indicated good and moderate evidence, respectively. “Good” scores were equivalent to more than two reviews, and “moderate” scores were equivalent to at least one non-systematic review. Finally, a net score of less than 4 was considered insufficient evidence and was not labeled.

**Table 4:** **Summary of scores and color codes for each level of evidence**

**Level of evidence**	**Range of scores**	**Code**
Strong evidence of positive effect	15 and above (+)	[+++]
Good evidence of positive effect	10-14 (+)	[++]
Moderate evidence of positive effect	4-9 (+)	[+]
Insufficient evidence	3.5 (−) to 3.5 (+)	[0]
Moderate evidence of negative or null effect	4-9 (−)	[−]
Good evidence of negative or null effect	10-14 (−)	[−−]
Strong evidence of negative or null effect	15 and above (−)	[−−−]

## Results

Abstractors identified a total of 521 results from 221 sources, coming from at least 17 countries. Four hundred eighteen of these results were from a source with a quality score of at least “3” and were used in the results reported here. The five setting-specific tables with notes about each study and codes for findings are available online (Additional file 1).

### Open spaces/parks/trails

Table 5 summarizes 69 findings from studies in the open space/parks/trails setting. Of the 36 cells representing attribute by co-benefit combinations in the table, 3 had strong evidence of co-benefits, 3 had good evidence, and 7 had moderate evidence. There was good to strong evidence for the association between park presence/proximity and all co-benefits, except for economic benefits. Moderate evidence supported that physical activity promotion programs in parks and open spaces were associated with four co-benefits of mental health, social benefits, environmental benefits, and safety/injury prevention. Public gardens had moderate evidence of social and safety/injury prevention benefits. There was good evidence that trails had economic benefits. Overall, there were 23 “blank cells” out of 36, indicating no or insufficient evidence.

**Table 5:** **Open spaces/parks/trails summary scores**

**Built environment attribute**	**Physical health**	**Mental health**	**Social benefits**	**Environmental sustainability**	**Safety/injury prevention**	**Economic benefits**
**Presence, proximity**	[+++] 54 + 3.5(0)	[+++] 88.5+	[+++] 26.5 + 4(0)	[++] 16 + 4(0)	[++] 11+	[0] 7.5 + 4(0)
**Design features**	[0] 3.5+		[+] 7.5+			
**Trails**						[++] 11.5+
**Physical activity programs/promotion**		[+] 4.5+	[+] 4+	[+] 4+	[+] 4+	
**Incivilities**					[0] 3.5+	
**Public gardens**			[+] 4.5+		[+] 4.5+	

### Urban design

There were 202 findings used in the summary of the urban design setting. Of 30 cells (Table 6), 8 had strong evidence of co-benefits, 5 had good evidence, and 6 had moderate evidence. In the urban design setting, 4 cells had moderate or good evidence of negative effects and one cell had strong evidence for negative effects, which was mixed use and safety/injury prevention. Mixed use, greenery, street scale design, and accessibility and street connectivity had evidence of 4 to 5 co-benefits. With the exception of street-scale design, urban design features had strong evidence of environmental benefits. All urban design features had evidence of economic benefits, and the evidence was particularly strong for mixed use. Of all urban design features, only greenery had strong evidence of mental health benefits. None had evidence of safety/injury prevention benefits. Residential density had the most complex pattern, with good evidence of negative health effects, strong evidence of environmental sustainability benefits, and good evidence of economic benefits.

**Table 6:** **Urban design summary scores**

**Built environment attribute**	**Physical health**	**Mental health**	**Social benefits**	**Environmental sustainability**	**Safety/injury prevention**	**Economic benefits**
**Residential density**	[−−] 19 + 21.5(0) 7.5-		[−] 13.5 + 14.5(0)	[+++] 88 + 21(0) 3.5-	[−−] 4.5(0) 7.5-	[++] 15 + 3.5(0)
**Mixed land use**	[+] 28 + 17(0) 4-	[0] 4.5 + 4-	[+++] 33 + 11(0)	[+++] 95 + 21(0)	[−−-] 4.5(0) 11-	[+++] 22.5 + 3.5(0) 4-
**Streetscale pedestrian design**	[+] 7.5+		[+] 7.5+	[+] 7.5+		[+] 7+
**Greenery**	[+++] 20.5 + 3.5(0)	[+++] 26.5+	[++] 12+	[+++] 39.5+		[++] 12+
**Accessibility & street connectivity**	[++] 30 + 12(0) 7.5-		[++] 14.5 + 3.5(0)	[+++] 35.5 + 3.5(0)	[−] 4.5(0)	[+] 12.5 + 3.5(0)

### Transportation systems

There were 81 findings in the transportation systems category. Of 48 cells (Table 7), 5 had strong evidence of co-benefits, 2 had good evidence, and 6 had moderate evidence. Strong evidence of co-benefits was most apparent in the safety/injury prevention and economic domains. Pedestrian and bicycle facilities had the best evidence of multiple co-benefits, followed by lower traffic speed and volume. Public transport had strong evidence of economic benefits and mixed evidence of environmental sustainability benefits. Overall, 34 of 48 combinations of environmental feature and co-benefit had no or inadequate evidence, showing many research gaps.

**Table 7:** **Transportation systems summary scores**

**Built environment attribute**	**Physical health**	**Mental health**	**Social benefits**	**Environmental sustainability**	**Safety/injury prevention**	**Economic benefits**
**Pedestrian/bicycle facilities**		[0] 3+	[+] 7+	[+] 10.5 + 3.5(0)	[+++] 27.5 + 4(0)	[+++] 22.5 + 3.5(0)
**Crosswalk markings**					[−−] 6(0) 4-	
**Traffic calming**	[0] 3.5+	[0] 3.5(0)	[0] 3+	[0] 3 + 3-	[+++] 23+	[0] 3+
**Public Transportation**	[0] 3.5-			[++] 28.5 + 17.5(0)		[+++] 20 + 4-
**Traffic speed/volume**	[0] 3.5+		[0] 3+	[+++] 14+	[+] 7+	[+] 7+
**Safe routes to school**			[0] 3+	[0] 3.5+	[+] 9.5 + 4(0)	
**Ciclovia/play streets**			[+] 7+			[0] 3.5+
**Managed parking**				[++] 10.5+		

### Schools

There were 27 findings in the school setting category. Of the 18 cells in Table 8, two cells had strong evidence of co-benefits, one had good evidence, and five had moderate evidence. Siting schools near the homes of students had strong evidence of environmental sustainability benefits and moderate evidence of mental health and economic benefits. Recreation facilities at schools and shared use agreements had evidence of multiple co-benefits.

**Table 8:** **Schools summary scores**

**Built environment attribute**	**Physical health**	**Mental health**	**Social benefits**	**Environmental sustainability**	**Safety/injury prevention**	**Economic benefits**
**School siting**	[0] 3.5+	[+] 4.5+		[+++] 21.5+	[0] 3-	[+] 4+
**Recreation facilities**	[++] 16 + 3.5(0)	[+++] 16.5+	[0] 3.5+			[0] 3.5+
**Shared use agreements**			[+] 7.5+		[+] 4+	[+] 7.5+

### Workplaces/buildings

There were 39 findings in the workplace/building category. Of the 36 cells in Table 9, three cells had good evidence of co-benefits and three had strong evidence. Specifically, building site design (mainly outdoor) features had strong evidence of physical and good evidence of mental health benefits, and features of the building design had strong evidence of physical health and good evidence of environmental sustainability and economic benefits. Physical activity programs and policies within workplaces had strong evidence of economic benefits. For workplace and building features, the best evidence was for physical health and economic benefits.

**Table 9:** **Workplaces/buildings summary scores**

**Built environment attribute**	**Physical health**	**Mental health**	**Social benefits**	**Environmental sustainability**	**Safety/injury prevention**	**Economic benefits**
**Building siting**	[+] 4+					
**Mixed land use around worksite**				[+] 4+		[+] 4+
**Building site design**	[+++] 16+	[++] 11.5+				[0] 3.5+
**Building design**	[+++] 19.5+	[0] 3.5 + 4-		[++] 12.5+		[++] 12+
**Worksite physical activity policies and programs**	[+] 8.5+	[0] 3.5+		[+] 4+		[+++] 25+
**Workplace furniture design**	[0] 7 + 3.5(0)					[0] 3.5 + 3.5(0)

### Overall summary of co-benefits by setting

In the final table, results were summed across features for each of the five settings. These results are intended to illustrate the overall potential for each setting to contribute to each co-benefit. Table 10 represents 418 findings. Of the 30 cells in the matrix, 22 had strong evidence of co-benefits and 2 had good evidence. Five cells had inadequate evidence, and only one cell had evidence of a net negative effect. Open spaces/parks/trails was the only setting with good to strong evidence of all six co-benefits. Activity-friendly design features in all five settings had strong evidence of environmental and economic benefits. Many gaps in the evidence existed for the transportation and workplace/buildings settings, particularly regarding the outcome of safety/injury prevention. There was little evidence of negative consequences of activity-friendly environments. However, in the urban design setting there was some evidence of negative physical health and safety/injury outcomes, mainly related to high residential density.

**Table 10:** **Overall co-benefits by setting summary scores**

**Built environment attribute**	**Physical health**	**Mental health**	**Social benefits**	**Environmental sustainability**	**Safety/injury prevention**	**Economic benefits**
**Open spaces/Parks/Trails**	[+++] 57.5 + 3.5(0)	[+++] 93+	[+++] 42.5 + 4(0)	[+++] 20 + 4(0)	[+++] 23+	[+++] 19 + 4(0)
**Urban design**	[+++] 105 + 54(0) 19-	[+++] 31 + 4-	[+++] 80.5 + 29(0)	[+++] 265.5 + 45.5(0) 3.5-	[−−−] 13.5(0) 18.5-	[+++] 69 + 10.5(0) 4-
**Transportation systems**	[0] 7 + 3.5-	[0] 3 + 3.5(0)	[+++] 23+	[+++] 70 + 21(0) 3-	[+++] 67 + 14(0) 4-	[+++] 56 + 3.5(0) 4-
**Schools**	[+++] 19.5 + 3.5(0)	[+++] 21+	[++] 11+	[+++] 21.5+	[0] 4 + 3-	[+++] 15+
**Workplaces/Buildings**	[+++] 55 + 3.5(0)	[++] 18.5 + 4-		[+++] 20.5+		[+++] 48 + 3.5(0)

## Discussion

The present exploration of diverse peer-reviewed and gray literature revealed substantial documentation that designing communities that support physical activity for both recreation and transportation purposes is likely to produce a wide variety of additional benefits, ranging from mental health to environmental sustainability and economics. The present paper is the first attempt to compile such a wide range of evidence, and the results supported multiple potential co-benefits of designing environments for active living. This initial synthesis can guide future research and can serve as an interim tool for evidence-based decision-making regarding planning and design of built environments. A longer report version of the present study, with additional detailed information about methods, findings and additional sections on disparities and policy implications is presented online (Additional file 2).

When the results from all features were combined, there was impressive evidence of co-benefits in all settings, with 22 of 30 cells having strong evidence. For all settings, there was strong evidence for at least three of the six co-benefits of activity-friendly design. Within each setting there were several features that could be designed to create activity-friendliness. Thus, the present paper can be viewed as a menu of options that would allow designers and planners to devise multiple combinations of features to achieve activity-friendly environments. If there was strong evidence that a feature in a setting was related to the co-benefit, then designing this feature to support physical activity might yield the co-benefits indicated by this review.

There is mounting evidence that multiple environmental features or patterns of features combine to produce stronger effects on physical activity than any single feature [25,40,41], and this principle may apply to the co-benefits as well. If several features of a setting had relatively weak evidence of co-benefits, it is reasonable to expect that optimizing these multiple features could, in aggregate, produce strong effects. Thus, it is justified to sum the co-benefits scores across multiple features to estimate the potential overall effect of designing a setting to optimize physical activity, as was done to create Table 10.

The most studied setting was urban design, with more findings reviewed than all other settings combined. All features examined, including high residential density, mixed land use, activity-friendly street scale design, greenery, and high accessibility and street connectivity, were associated with environmental and economic benefits. In contrast, much less evidence was identified for the schools and workplaces/buildings settings. Blank cells in the summary tables indicate gaps to be filled by future research. For example, programs such as “Safe Routes to School” and Ciclovias or Open Streets may have numerous benefits, such as improved social benefits, reduced carbon emissions, and cleaner air, but these outcomes remain to be documented.

One notable finding was that economic benefits of activity-friendly designs were documented for all five physical activity settings. Based on the specific studies identified, many groups could enjoy economic benefits of activity-friendly environments, including governments (due to reduced spending on infrastructure), homeowners, real estate developers, health insurance companies, employers, retailers, commercial property owners, and taxpayers. This is an extremely broad range of beneficiaries, and some of them may not be aware of the economic benefits of activity-friendly environments.

### Policy implications

Policy-makers worldwide are faced with many problems and challenges [42]. Rates of chronic disease and related costs are high in countries at all income levels, and these rates are increasing fastest in low- and middle-income countries [4]. Depression creates the highest burden of disease worldwide [43], and injuries are the biggest cause of death among young people [43]. The consequences of climate change are expected to be the worst human-made disasters in history [44]. Every country and city is looking for ways to improve economic growth. It seems inconceivable that making cities better for physical activity could contribute to solutions of all these problems. However, the evidence compiled here suggests designing activity-friendly communities could be a partial solution for many critical problems.

If decisions about built environments were informed by evidence, then the features with the best evidence of co-benefits listed in Table 11 would deserve special consideration. The features listed in Table 11 had at least “moderate” evidence of three co-benefits. Among these, the best supported environmental features with at least “good” evidence of three co-benefits were park proximity, mixed land use, greenery, accessibility and street connectivity, building design, and workplace physical activity policies/programs.

**Table 11:** **Best evidence of environmental features with strong multiple benefits (at least “moderate” evidence of three benefits)**

**Setting**	**Built environment attribute**	**Evidence**
Open Spaces/Parks/Trails	Park presence/proximity	3 strong, 2 good
	Programs, promotion, and events	4 moderate
Urban Design/Land Use	Mixed land use	3 strong, 1 moderate (1 strong negative)
	Greenery	3 strong, 2 good
	Streetscale pedestrian design	4 moderate
	Accessibility and street connectivity	1 strong, 2 good, 1 moderate (1 good evidence of negative)
Transportation	Pedestrian/bicycle infrastructure	2 strong, 2 moderate
	Reduced traffic speed and volume	1 strong, 2 moderate
Schools	School siting	1 strong, 2 moderate
	Shared use agreements	3 moderate
Buildings/Workplaces	Building design	1 strong, 2 good
	Physical activity policies and programs	1 strong, 2 good

### Strengths and limitations

The strength of the literature exploration was the breadth of topics explored. For each setting several features were identified that were related to physical activity, and each of these features was evaluated for six types of co-benefits. A quasi-quantitative approach was used to code the level of evidence of 521 findings and weight each finding by the quality of the source. To avoid basing findings on lower quality evidence, such as poorly-substantiated claims in advocacy documents, lower-quality evidence was not included in the numerical summaries.

The main limitations were a consequence of the breadth. Because of the large number of topics searched, it was not possible to conduct a systematic review. A requirement of systematic reviews is assessment of the quality of each study, but this was not feasible given the number and diverse types of studies. It would have been helpful to code studies as being cross-sectional, longitudinal, or experimental in design. Existing reviews were used whenever possible to reflect the best evidence in the literature. Literature searches and coding were conducted by several investigators with a semi-structured process. Therefore there were undoubtedly differences across topics in thoroughness of search and classification of levels of evidence.

The searches were limited to English language documents, but one-quarter of the findings were from countries other than the US, and another quarter of findings were from reviews that included international literature. Another limitation was publication bias that favors positive findings, though this may have been countered somewhat by inclusion of gray literature, including technical reports. The intent of the literature exploration was to identify as many relevant sources as possible to determine whether it is worthwhile to pursue the topic of co-benefits, but a weakness was that the quality of each source was merely categorized and not based on an analysis of methodological quality.

The summary scores are not intended to be interpreted literally as the actual strength of evidence, but they provide a rough indication of the extent of evidence: pro, con, and neutral. If the net scores for the level of evidence are strong, there is reason to have confidence in the finding for a connection between a feature and an outcome because a strong rating required findings from multiple sources.

## Conclusions

Substantial evidence indicated that designing and creating parks, communities, transportation systems, schools, and buildings that make physical activity attractive and convenient is also likely to produce a wide range of additional benefits. Present findings provide new information to decision-makers in numerous sectors that could change the perceived benefits of activity-friendly designs. Benefits were found for environmental sustainability, economics, and multiple dimensions of health. Though the present review is not definitive, the large number of sources identified for such policy-relevant issues provides a compelling justification for more original research and systematic reviews of the very broad range of topics. If “a good solution solves multiple problems,” then building places that support physical activity may be considered a superlative solution.

## Additional files

Additional file 1:
**Detailed Information About Each Study Coded.** A spreadsheet of the five setting-specific tables with notes about each reviewed report/study and codes for findings so sources and interpretations are transparent to reviewers and readers. Available on the Active Living Research website [http://activelivingresearch.org/making-case-designing-active-cities]. Additional file 2:
**Making the Case for Designing Active Cities.** A longer report version of the present study, with additional sections, available on the Active Living Research website [http://activelivingresearch.org/making-case-designing-active-cities]. The report gives more details on the methods and findings as reported in the manuscript and includes additional sections on disparities and public opinion on active living environmental features.
