# Supplementary material for: Co-benefits of designing communities for active living: an exploration of literature
Source: Int J Behav Nutr Phys Act. 2015 Feb 28;12:30. doi: 10.1186/s12966-015-0188-2 (PMC4349686; doi:10.1186/s12966-015-0188-2)
Supplement: Additional file 2: — Making the Case for Designing Active Cities. A longer report version of the present study, with additional sections, available on the Active Living Research website [http://activelivingresearch.org/making-case-designing-active-cities]. The report gives more details on the methods and findings as reported in the manuscript and includes additional sections on disparities and public opinion on active living environmental features. [file 12966_2015_188_MOESM2_ESM.pdf]

Making the Case for Designing Active Cities

This report was prepared by:

James F. Sallis, PhD, Active Living Research, University of California, San Diego
Chad Spoon, MRP, Active Living Research, University of California, San Diego

Contributors:

Nick Cavill, PhD, MPH, MFPH, Cavill Associates Ltd, United Kingdom
Jessa Engelberg, BA, UCSD / SDSU Public Health Joint Doctoral Program
Klaus Gebel, PhD, James Cook University, Australia
Debbie Lou, PhD, University of California, San Diego
Mike Parker, BSc, Progress Health Partnerships, United Kingdom
Christina M. Thornton, JD, MA, UCSD / SDSU Public Health Joint Doctoral Program
Amanda Wilson, MSRS, Active Living Research
Carmen L. Cutter, MPH, Active Living Research
Ding Ding, PhD, MPH, The University of Sydney, Australia

For a Web-based version of this report, visit:
http://activelivingresearch.org/making-case-designing-active-cities

A peer-reviewed paper based on this report is available online through open access:

Citation and URL coming soon.

TECHNICAL REPORT
Initial Version, June 2014
Revision, February 2015

ACTIVE LIVING RESEARCH

TABLE OF CONTENTS

Executive Summary 3
Introduction 4
Making the Case Methodology 4
Results of Reviews 9
Open Spaces / Parks / Trails 11
Urban Design / Land Use 13
Transportation Systems 15
Schools 18
Buildings / Workplaces 20
Review of Grey Literature in Europe 22
Special Report on Active Living and Climate Change 24
Conclusions ……. 26
Recommendations 30
References ……. 32

ACKNOWLEDGEMENTS

This project was supported by Nike, Inc and Active Living Research, a program of the Robert Wood Johnson Foundation. Thanks to the content experts from multiple disciplines and sectors who provided input on the review process.

Executive Summary

Creating "activity-friendly environments" is recommended to promote physical activity, but potential co-benefits of such environments have not been well described. An extensive but non-systematic review of scientific and "gray" literature was conducted to explore a wide range of literature to understand the co-benefits of activity-friendly environments on physical health, mental health, social benefits, safety/injury prevention, environmental sustainability, and economics. Five physical activity settings were defined: parks/trails, urban design, transportation, schools, and workplaces/buildings.

KEY FINDINGS

- A total of 418 higher-quality findings were summarized based on direction of association and quality of source.
- The overall summary indicated 22 of 30 setting by outcome combinations showed “strong” evidence of co-benefits.
- Each setting had strong evidence of at least 3 of the 6 co-benefits, and parks and trails had strong evidence of all 6 co-benefits. Thus, for each setting there are multiple features that can be designed to both facilitate physical activity and produce co-benefits.
- All five physical activity settings could be designed so they have positive effects on economic outcomes, including increased home value, greater retail activity, reduced health care costs, and improved productivity.
- Activity-friendly design in all settings had strong evidence of environmental co-benefits based on reduced pollution and carbon emissions.
- There were many gaps in evidence of co-benefits in the schools and workplace settings as well the health consequences of environments that support active travel.
- Overall, there was little evidence of negative consequences of activity-friendly environments.

IMPLICATIONS

The most important conclusion of this review is that creating communities, transportation systems, schools, and buildings that make physical activity attractive and convenient also produces a wide range of other benefits for communities. Rather than thinking that designing one feature of a transportation system or school is sufficient, we encourage decision-makers and designers to consider how features in all settings can be optimized for physical activity and multiple other benefits. We urge mayors, other city officials, and staff in multiple departments to consult these findings as an aid in decision-making.

Introduction

As demonstrated in Designed to Move (www.designedtomove.org), physical inactivity is a major challenge to health and well-being globally. There are 5 million deaths worldwide attributable to physical inactivity (Lee, et al, 2012), most people are not sufficiently active, physical activity is declining in many countries, and this is a global problem with the biggest burden in low and middle income countries (Ng & Popking, 2012). Increasing physical activity is a goal of the United Nations through its non-communicable disease initiative (UN, 2011).

Physical activity has been engineered out of people's lives, but efforts are underway worldwide to re-integrate physical activity into daily lives through environmental changes. The UN, World Health Organizations, national physical activity plans, US Centers for Disease Control and Prevention, US Institute of Medicine, and other scientific groups worldwide have identified creating built environments and implementing policies that support active living as essential for increasing physical activity and improving health.

There are special opportunities to create more activity-friendly environments through actions taken by city governments. Actions at the city level can affect urban design, transportation systems, parks and recreation facilities, schools, and workplaces/buildings. These actions are often led by mayors. Thus, we look to mayors for leadership in creating activity-friendly environments, and mayors around the world are showing leadership and achieving results.

An important barrier to environmental changes that support physical activity is that decision-makers do not consider physical activity an important enough rationale to justify policy changes and investments. Even if a decision-maker is well aware of the important effects of physical activity on health and health care costs, health may not be on the agenda of the decision-maker. Decisions taken in the transportation, city planning, parks and recreation, education, architecture, and business fields often determine whether environments facilitate, or create barriers to, physical activity. Health is often not part of the mission of these groups. Though mayors, city councilors, and similar officials work everyday to balance competing interests, most of them do not understand that environments that support physical activity produce additional benefits for their communities. Thus, creating activity-friendly environments can be a solution that solves multiple problems. For example, changing zoning codes to favor mixed use developments is likely to enhance property values and reduce carbon emissions. Having parks in neighborhoods has been linked with physical health and mental health benefits.

However, there is no resource that describes the full range of co-benefits of activity-friendly environments. We undertook an exploration of an extremely diverse literature ranging from mental health and injury prevention to real estate prices and climate change. We searched in the scientific literature and beyond. We asked for guidance from scholars and advocates working in diverse fields. We looked hard for international evidence and dug into "green" benefits that related to pollution and carbon emissions. The hunt uncovered hundreds of relevant studies and reports that can be used to create a profile of the multiple benefits of each environmental feature. We believe this report can be a useful decision tool. Because mayors and city councilors must address a very broad set of issues, knowing how a single decision can affect several outcomes may change that decision. Built environment decisions often involve large financial commitments, and they create the form and character of cities that last for decades, even centuries. Decisions about built environments should consider the full range of consequences, including physical activity, and this report is meant to be a tool for decision-makers.

The main audience for this report is mayors, city officials, practitioners from multiple sectors, and advocates. The goal is to summarize the available science in a non-technical way so non-scientists can use the evidence in their decision-making.

Making the Case Methodology

Active Living Research's (ALR) primary task was to identify physical activity-related built environment features, then conduct a literature review to examine multiple potential benefits across a wide range of sectors, including chronic diseases, injury, mental health, social benefits, economic benefits, automobile congestion, air pollution, and carbon emissions. Thus, the focus was on potential benefits of each built environment feature, in addition to physical activity. The findings of the literature review were summarized in tables, figures, and quotes to aid the reader in understanding the level of evidence for multiple benefits of built environment features that can support physical activity

We first developed a list of potential outcomes or co-benefits of activity-supportive built environments (Table 1). With guidance from international experts convened at a September 2013 Active Cities workshop, we then identified built environment features from each of the five Designed to Move (DTM) settings that research had shown to be related physical activity (Table 2). We used these built environment features to structure and narrow the scope of the literature search.

Table 1: Outcomes of Activity Supportive Built Environments

OUTCOME / CO-BENEFIT	DESCRIPTION
Physical health	Chronic diseases, obesity
Mental health	Depression, anxiety, well being, quality of life
Social benefits	Neighborhood/social cohesion, human capital
Environmental benefits	Carbon dioxide emissions, pollutants
Injury prevention	Crime, violence, car crashes
Economic benefits	Land value, governmental infrastructure costs, real estate profitability, productivity/job performance, health care costs, economic performance of cities
Other	Automobile congestion, findings related to disparities, polls showing public support or opposition to an environmental feature

Table 2: Built Environment Features Correlated with Physical Activity

BUILT ENVIRONENT ATTRIBUTE	DESCRIPTION	REFERENCE
**Open Spaces / Parks / Trails**		
Design features	Size, amenities/facilities	Godbey, 2010; Ding, 2011; Sallis, Adams, 2011
Presence/proximity	Existence of and distance to	Ding, 2011; Bauman, 2012; Sallis, 2012
Trails	Proximity to and design of	Sallis, Adams, 2011; Sallis, 2012
Programs, promotion, and events	Park-based programming	Godbey, 2010
Park incivilities / civilities	Existence of or lack of graffiti, litter, anti-social behavior (public drinking, loitering)	Molnar, 2004; Miles, 2008; Sallis, Adams, 2011
Public gardens		Godbey, 2010
**Urban Design / Land Use**		
Density	Population and housing density	Ding, 2011; Bauman, 2012; Sallis, 2012
Mixed land use	Mix of destinations, distance to destinations	Ding, 2011; Bauman, 2012; Sallis, 2012
Streetscale pedestrian design	Including buffers, building set-back, form based codes, street lights, etc	Heath, 2006
Greenery	Street trees/shrubbery, gardens	Ding, 2011;<br>Bauman, 2012
Incivilities	Graffiti, vacant/dilapidated buildings, litter, anti-social behavior (public drinking, loitering)	Molnar, 2004; Miles, 2008; Ding 2011
Accessibility and connectivity	Street network	Ding, 2011; Sallis, Adams, 2011; Bauman, 2012

**Transportation Systems**		
Pedestrian/bicycle infrastructure	Sidewalks, bike lanes, bike parking	Ding, 2011; Bauman, 2012; Sallis, 2012
Crosswalk markings	Crosswalks (intersections as its own location)	Badland, 2005
Traffic calming	Speed bumps, curb-cuts, road diet, other engineering infrastructure	Badland, 2005; Rothman, 2014
Public transportation	Bus, train	Sallis, Adams, 2011; Bauman, 2012; Sallis, 2012
Traffic speed/volume		Ding, 2011
Safe routes to school	Engineering, programming, promotion and events	Sallis, 2012; Stewart, 2014
Ciclovia/play streets	Open streets	Sarmiento, 2010; Torres, 2013
Managed parking	Parking access	U.S. EPA, 2006
**Schools**		
School siting	Location of school (suburban, urban, rural)	McMillan, 2009
Recreation facilities	Physical education facilities and equipment, presence of PE teachers	Davison, 2006; Spengler, 2012
Shared use agreements	Community use of school facilities	Spengler, 2012; Lafleur, 2013
**Workplaces / Buildings**		
Building siting	Access to active transportation opportunities	Zimring, 2005
Mixed land use around worksite	Mix of destinations, distance to destinations	Ding, 2011; Bauman, 2012; Sallis, 2012
Building site design	Design of property that building sits upon	Zimring, 2005
Building itself	Stair design, exercise equipment presence, shower/locker presence, skip-stop elevators	Zimring, 2005; Nicoll, 2009
Worksite physical activity policies and programs	Exercise classes, discounted gym membership, active transportation promotion policies, parking cash out programs, point-of-decision prompts	Proper, 2003; Crespo, 2011
Workplace furniture design	Sit-stand desks	Alkhajah, 2012

Literature Review

We began the literature review with a snowball sampling of topic experts. Using our ALR network of diverse experts, we asked 20 leaders with expertise in the five DTM settings for their help in identifying:

- Groups/organizations working on these issues worldwide
- Must see reports/papers, both peer-reviewed and grey literature
- Websites
- Cities that have implemented activity-friendly built environment changes
- Recommendations for other experts in the field we should speak with

Of the 20, we received input from 13 experts (Table 3). From November 2013 through February 2014, ALR staff reviewed the recommended resources, looking for evidence focused on our identified built environment features and co-benefits. Some of the recommended resources focused specifically on physical activity as an outcome and were disqualified from the abstracting. ALR staff used the recommended resources to find additional papers and reports using the citation lists. Both peer-reviewed and "gray" literature and reports were used. To fill gaps, staff also performed

targeted literature searches using the built environment features and co-benefits as search terms to uncover additional resources. However, systematic literature reviews designed to identify all relevant papers were not feasible given the large scope of the search. Abstractors were instructed to use multiple synonyms for search terms because terms vary by discipline. Search engines included Scopus, PubMed, Google Scholar, ISI Web of Science, MEDLINE, PsychINFO, Academic Search Premier, ClimateArk, and Google.

Table 3: Topic Experts Providing Input

NAME	AFFILIATION
**Open Spaces/Parks/Trails**	
Ariane Rung, PhD, MPH	Louisiana State University, School of Public Health
Andy Mowen, PhD	The Pennsylvania State University, Department of Recreation, Park and Tourism Management
Zarnaaz Bashir, MPH	National Recreation and Park Association, Health Initiatives
Karla Henderson, PhD	North Carolina State University, Department of Parks, Recreation, and Tourism Management
**Urban Design/Land Use**	
Anne Vernez Moudon, Dr es Sc	University of Washington, Department of Urban Design & Planning
Andy Dannenberg, MD, MPH	University of Washington, School of Public Health
Reid Ewing, PhD	University of Utah, Department of City & Metropolitan Planning
**Transportation**	
Robert Cervero, PhD	University of California, Berkeley, Department of City & Regional Planning
Charlie Zegeer, MS	University of North Carolina Highway Safety Research Center
Chris Kochtitzky, MSP	Centers for Disease Control and Prevention, Division of Emergency & Environmental Health Services
**Schools**	
Jeff Vincent, PhD	University of California, Berkeley, Center for Cities & Schools
Nisha Botchwey, PhD	Georgia Institute of Technology, School of City & Regional Planning
**Buildings/Workplaces**	
Gayle Nicoll, PhD	Ontario College of Art & Design, Faculty of Design

Abstracting Process

During the abstracting process, basic information on the built environment feature, co-benefit, study sample characteristics, study methods, and major research findings were listed in tables specific to each DTM setting (see Excel spreadsheet titled “Data Abstracting Table” at http://activelivingresearch.org/making-case-designing-active-cities). Staff graded the scientific rigor of each piece of evidence (Table 4) and noted the direction of association of the benefit with the environmental feature (Table 5). To simplify interpretation, "+" denotes an environmental feature is associated with a co-benefit in a "favorable" direction, which could be better mental health or fewer carbon emissions. As much as possible, quantitative findings were included so as to offer specificity in terms of impact of the built environment feature on the co-benefit. ALR staff were instructed to also look for findings relevant to international examples, disparities by income or race/ethnicity, and public opinion polling to include in the abstracting process. Public opinion polling was included because such data are relevant to decision-making by government leaders. However, it was not feasible to grade the quality of the studies, as is done in systematic reviews.

Table 4: Grading the Evidence for Resource Abstracting Process	
**SCORE**	**TYPE OF EVIDENCE**
4.5	Peer-reviewed, systematic review paper (including meta-analysis)
4	Peer-reviewed, non-systematic review paper (from scientific literature) or non-peer-reviewed review paper (from grey literature)
3.5	Any (singular) peer-review study
3	Any (singular) non peer-reviewed study, such as a technical report from a government agency or academic center
2	Non-analytic studies (for example, case reports, case series, simulations) or advocacy report without a clear literature review
1	Expert opinion, formal consensus

Table 5: Direction of Association of Evidence for Resource Abstracting Process	
**SCORE**	**ASSOCIATION**
+	A favorable association was found between feature and co-benefit (feature associated with "better" level of co-benefit
-	An unfavorable association was found between feature and co-benefit (feature associated with "worse" level of co-benefit
0 (zero)	No association or inconsistent evidence was found between feature and co-benefit

The initial goal was to be inclusive in finding relevant sources of information. For scientific literature, reviewers were instructed to find systematic or non-systematic reviews first. If reviews were located, then the individual studies did not need to be searched, except for publications since the latest review. In cases where a review paper did not provide adequate specificity or quantification in the findings, selected primary studies from that review paper were abstracted to illustrate specific findings. If reviews were not located, then individual studies were searched. For gray literature, reports from credible organizations were targeted, from such groups as government agencies, academic centers, and selected advocacy groups. Newspapers, magazines, and blogs were not searched, except to identify citations of or links to more credible reports. Abstracting tables were cross-checked by other staff for accuracy and clarity.

To ensure a global perspective, ALR contracted with two international consultants to replicate the literature review process as well as provide additional expertise. Nick Cavill, PhD and Mike Parker of Cavill Associates, a public health consultant firm based in the United Kingdom, conducted a search of grey European literature, uncovering and abstracting a total of 46 reports. Ding Ding, PhD, MPH of the University of Sydney and Klaus Gebel, PhD of James Cook University, Australia, conducted a literature search focused on the association between the built environment features and climate change related outcomes, and case studies of successful programs, policies, or planning initiatives that were successful in reducing carbon emissions. Ding and Gebel added a total of 31 reports to the overall MTC abstracting.

Synthesizing the Findings

On completion of the abstracting process, ALR staff and the international consultants selected ‘key findings’ that they judged to be good examples for illustrating findings and providing specific quantitative results. These key findings were identified based on relevance and quality of the study or finding. The key findings are used as evidence and examples in this overall summary report.

Communicating so many findings is a challenge, so several ways of summarizing the results in a visual manner were created. To illustrate areas with strong research evidence as well as research gaps, ALR staff created summary matrices that visually show which built environment features have (and do not have) evidence supporting a co-benefit (see Table 6 for example; for full document, see Excel spreadsheet titled “Summary Matrix” at http://activelivingresearch.org/making-case-designing-active-cities. Each finding from the Data Abstracting tables is listed in the appropriate cell along with its associated grade of evidence and direction of association. Individual papers that were counted as part of a review paper, and therefore also abstracted, are coded in red on the Summary Matrix to help prevent double counting of findings. The Summary Matrix helps to identify specific policy and built environment changes that cities can pursue within a DTM setting.

An overall “Making the Case” summary table identifies which DTM settings have the most overall evidence. The table was calculated by aggregating the "grading the evidence" scores for each built environment feature for each co-benefit within each DTM setting (see Tables 8 - 12). Dark green cells have the highest levels of evidence, light green cells have moderate evidence, white cells lack sufficient evidence to make a judgment, and pink cells have evidence of unfavorable associations. Thus, decision-makers can see at a glance which environmental features have the best evidence of multiple benefits.

Table 6: Example of “Summary Matrix”

OPEN SPACES / PARKS / TRAILS

Feature	Physical Health	Mental Health	Social Benefits	Environmental Benefits	Injury Prevention	Economic Benefits	Opinion Polling	Disparities	Does Not Apply
Design features	50a: 3.5+		34: 4+; 49a: 3.5+						
Presence/proximity	3a: 3.5+; 5a: 3.5+; 6: 3.5(0); 7a: 3.5+; 8: 3.5+; 11a: 3.5+; 14: 3.5+; 17: 4.5+; 21a: 4.5+; 23a: 4.5+; 37: 4+; 46: 4+; 50b: 3.5+; 57a: 2-; 60a: 4+; 61a: 4+ [54+; 3.5(0)]	1: 4.5+; 3b: 3.5+; 4: 3.5+; 5b: 3.5+; 7b: 3.5+; 10a: 3.5+; 13a: 4.5+; 15: 3.5+; 18: 4.5+; 20: 4.5+; 21b: 4.5+; 22: 4.5+; 23b: 4.5+; 24: 4.5+; 25: 4.5+; 36: 3.5+; 38: 4+; 39: 4+; 43: 3.5+; 52: 2+; 55: 2+; 59: 4+; 60b: 4+; 61b: 4+ [79+]	2: 4.5+; 9a: 3.5+; 11b: 3.5+; 12: 3.5+; 13b: 4.5+; 16: 3.5+; 49b: 3.5+; 53: 2+; 58: 2+; 63: 4(0) [26.5+; 4(0)]	41: 4+; 44: 4+; 45a: 4+; 47: 4+; 51: 2+; 62: 4(0) [16+; 4(0)]	9b: 3.5+; 10b: 3.5+; 40: 4+ [11+]	28: 3.5+; 45b: 4+; 54: 2+; 64: 4(0) [7.5+; 4(0)]		11c: 3.5+; 13c: 4.5+; 54b: 2-	56: 4.5+
Trails						29: 2+; 30: 2+; 31: 2+; 35: 4+; 42: 3.5+; 65: 4+ [11.5+]			
Programs, promotin, events		19: 4.5+;	33a: 4+	33b: 4+	32: 4+				
Park incivilities / civilities					48: 3.5+				
Public gardens			26a: 4.5+; 27: 2+		26b: 4.5+; 27: 2+				

Results of Reviews

Creating Color-Coded Matrix Tables to Summarize Results for Each Setting

The color-coded "matrix" tables that follow summarize the evidence of co-benefits for each key feature. There is a separate table of features for each of the five built environment settings. Some of the features are programs or social environment attributes that can complement a favorable built environment or possibly compensate for an unfavorable built environment.

We used a "quasi-quantitative" approach to summarizing the results of the literature reviews. Summary scores were created by summing the weights of evidence from each resource. Thus, the summary scores roughly indicate both the quantity and quality of the evidence. Cells in each table are color coded based on three levels of summary scores. We judge a positive finding from a literature review to be moderate evidence, because if coders found a good-quality review, they did not necessarily continue searching for specific studies. We did not want to set the bar too high, because we expected many of these topics not to be well studied. Thus, one high quality study does not merit a summary of "moderate" evidence, but a good review is considered "moderate" evidence.

We calculated "net" scores by subtracting the weight of negative and zero scores from the weight of plus scores (Table 7). Thus, evidence of negative or null findings were counted against positive findings. The color-coded “matrix” tables can be interpreted as follows:

- "4+" net score indicates moderate evidence (light green), because this is equivalent to one non-systematic review.
- "10+" net score indicates good evidence (medium green), because this is equivalent to more than two reviews.
- "15+" net score indicates strong evidence (dark green), because this is equivalent to more than three reviews.
- A net score of less than 4 indicated insufficient evidence and was coded with a white background.
- The same rules were used to summarize negative and null findings, with net negative or null findings coded in shades of red.
- A white cell with no numbers indicated that we found no credible evidence on this topic.
- In creating the summary scores, resources with quality scores of "1" or "2" were not counted because they were not considered credible enough.

Table 7: Summary of Scores and Color Codes for Each Level of Evidence

LEVEL OF EVIDENCE	RANGE OF SCORES	COLOR CODE
Strong evidence of positive effect	15 and above (+)	
Good evidence of positive effect	10-14 (+)	
Moderate evidence of positive effect	4-9 (+)	
Insufficient evidence	3.5 (-) to 3.5 (+)	
Moderate evidence of negative or null effect	4-9 (-)	
Good evidence of negative or null effect	10-14 (-)	
Strong evidence of negative or null effect	15 and above (-)	

Summary of Results by Setting

There are 3 parts of the presentation of results for each setting.

1. Key results and interpretations for each table.
2. Matrix table, with color codes and numerical scores for positive, null, and negative findings.
3. Samples of compelling findings from the “Data Abstracting Table”, to give a flavor of the types of studies and in some cases, to quantify the strength of associations or effects. See the “Data Abstracting Table” for full citations of items listed as key highlights (available at http://activelivingresearch.org/making-case-designing-active-cities).

Open Spaces / Parks / Trails

There were 69 entries in the open space/parks/trails category. Of 36 cells in the matrix table (Table 8), 3 had strong evidence of co-benefits, 3 had good evidence, and 7 had moderate evidence. Specific findings include:

- Park presence/proximity had good to strong evidence of all co-benefits, except economic.
- Physical activity programs and promotions had moderate evidence for 4 co-benefits.
- Public gardens had moderate evidence of social and injury prevention benefits.
- Trails had good evidence of economic benefits.
- There are many gaps in research on co-benefits of all parks and trails features, except park proximity and physical activity programs and promotion.

Current evidence supports a conclusion that having a park nearby with substantial programs and promotion produces a wide range of health and environmental benefits beyond physical activity.

Table 8: Open Spaces / Parks / Trails Summary Scores

Built Environment Attribute	Physical Health	Mental Health	Social Benefits	Environmental Sustainability	Safety / Injury Prevention	Economic Benefits
**Presence, proximity**	54+<br>3.5(0)	88.5+	26.5+<br>4(0)	16+<br>4(0)	11+	7.5+<br>4(0)
**Design features**	3.5+		7.5+			
**Trails**						11.5+
**Physical activity programs/ promotion**		4.5+	4+	4+	4+	
**Incivilities**					3.5+	
**Public gardens**			4.5+		4.5+	

Highlights of Key Parks and Recreation Findings

1. **Physical Health**
   a. <u>Presence/Proximity</u>: Diabetic individuals taking 30-minute walks in a forest experienced lowered blood glucose levels far more than the same amount of time spent doing physical activity in other settings. The half-hour forest walks resulted in larger drops in blood glucose than three hours of cycling. (*NRPA Report, 2010*)
   b. <u>Presence/Proximity</u>: The risk of cardiovascular and respiratory disease mortality decreased with increasing green areas for males ($p < 0.001$), and was lowest for the greenest wards (cardiovascular disease: incidence rate ratio (IRR) 0.95, 95% CI 0.91-0.98; respiratory disease: IRR 0.89, 95% CI 0.83-0.96). Thus, males living in the greenest urban wards in the UK had a 5% lower risk of cardiovascular disease mortality and 11% lower risk of respiratory disease mortality than those in the least green wards. In contrast, no association with urban green space was found among females for cardiovascular and respiratory mortality. (*Richardson & Mitchell, 2010*)
   c. <u>Presence/Proximity</u>: In areas where 90% of the environment around the home is green, only 10.2% of the residents feel unhealthy, as compared with areas in which 10% of the environment is green, where 15.5% of the residents feel unhealthy. (*Maas, 2006*)

2. **Mental Health**
    a. Presence/Proximity: People living more than 1km away from a green space resulted as having 1.42 higher odds of experiencing stress than those living less than 300m from a green space. An analysis of the association between stress and visits to green spaces showed that respondents who do not report stress have 1.57 (95% CI 1.40–1.76) higher odds of visiting a green space at least a few days a week than those reporting stress. The results also showed that the more often respondents visited green spaces, the less stress they experienced. (*Stigsdotter, 2010*)
    b. Presence/Proximity: 8 studies found that separation from nature via modern living is detrimental to human development, health, and wellbeing and that regular contact with nature, such as provided by parks, is required for mental health. (*Maller, 2008*)
    c. Presence/Proximity: Scientists in the Netherlands examined the prevalence of anxiety disorders in more than 345,000 residents and found that people who lived in residential areas with the least green spaces had a 44 percent higher rate of physician-diagnosed anxiety disorders than people who lived in the greenest residential areas. The effect was strongest among those most likely to spend their time near home, including children and those with low levels of education and income. In addition, the prevalence of physician diagnosed depression was 33 percent higher in the residential areas with the fewest green spaces, compared to the neighborhoods with the most. (*NRPA Report, 2010*)
    d. Presence/Proximity: In one study, researchers found that a 20-minute walk in an urban park benefited concentration performance of children with ADHD. The performance boost was at least as large as the peak performance boost shown for two widely prescribed ADHD medications. Researchers aren't the only ones who have tracked these benefits. In a nationwide survey, parents of kids with ADHD rated after-school activities in outdoor settings as significantly more helpful than those not in these settings. (*NRPA Report, 2010*)

3. **Social Benefits**
    a. Presence/Proximity: Significant relation between both the percentage of green in 1 km ($p < 0.05$) and in 3 km ($p < 0.01$) radii around people's home and their feelings of loneliness. People with more green space in their living environment feel less lonely. (*Maas, 2009*)
    b. Presence/Proximity: Spending time in green outdoor common spaces is systematically related to stronger social integration and a stronger sense of local community. In a study of inner-city older adults in Chicago, Ill., USA, the total amount of variation in sense of local community explained by exposure to green common spaces was 5%. (*Kweon, 1998*)
    c. Design Features: As part of Mayor Michael Bloomberg's PlaNYC 2030 and a three-way partnership between the board of education, the department of parks and recreation and The Trust for Public Land, New York City is rebuilding derelict schoolyards for students and opening them to the full community outside school hours. Each playground renovation begins with an in-depth participatory design phase and includes a 3-month student design phase. Following reconstruction of playgrounds, observations revealed a 25% increase in structured games and competition and a 240% increase in unstructured play. Unstructured activity is valuable in helping less athletic children enjoy recreation and develop social skills and imagination. (*Harnik, 2011*)

4. **Environmental Benefits**
    a. Presence/Proximity: Carbon storage and annual removal by urban park trees and soils in the United States is estimated at about:
    • Carbon storage (trees): 75 million tons ($1.6 billion)
    • Carbon storage (soils): 102 million tons of carbon ($2.1 billion)
    • Annual carbon removal (trees): 2.4 million tons ($50 million) (*Nowak & Heisler, 2010*)
    b. Presence/Proximity: Urban trees in the lower 48 states are estimated to remove 783,000 tons of pollution per year. *(NRPA Report, 2010*)
    c. Programs, promotion, events: Automobile traffic reduces park use. According to careful attendance counts by the San Francisco County Transportation Authority and extrapolations by the Center for City Park Excellence, offering a car-free park road on only one day of the week (Sunday) lures as many as 2.7

million more visitors annually to Golden Gate Park. A calculation by the Center for City Park Excellence indicates that closure of John F. Kennedy Drive seven days a week could increase total park usership by 69%, from about 12 million uses to 20 million uses a year. (*Harnik, 2011*)

5. **Economics**
   a. Presence/Proximity: The value of an average home increases with proximity to neighborhood parks, special parks, lakes, and rivers, with benefits ranging from a low of 0.0035% of sales price for every one percent decrease in the distance to the nearest neighborhood park, to a high of 0.0342% for every one percent decrease in the distance to the nearest lake. These effects are statistically significant at or near the 1% level. (*Anderson, 2006*)
   b. Trails: Public investments in the Platte River Greenway, Denver totaling about $70 million has fueled $2.5 billion in residential, commercial, retail, sports and entertainment projects along the corridor. (*Harnik, 2011*)
   c. Trails: On average about 30 percent of gross revenues were attributed to The Great Allegheny Passage trail (higher than the 25.5% reported in the 2008-2009 survey). (*Great Allegheny Passage Survey Report, 2012*)

6. **Injury Prevention**
   a. Programs, promotions, events: In Kansas City, crime in Kessler Park decreased by 74% the year that 2.6 miles of Cliff Drive were turned car-free on weekends. (*Harnik, 2011*)
   b. Presence/Proximity: In a study conducted in an inner-city Chicago low-rise apartment development, building with more vegetation were associated with: lower rates of homicide, assault, robbery, theft, burglary, and arson. Buildings with high levels of vegetation had 52 percent fewer total crimes than buildings with low levels of vegetation. (*NRPA Report, 2010*)
   c. Public Gardens / Park Incivilities / Civilities: 8 studies found that community gardens increase community cohesion, reduce graffiti and violence and enhance self-image of residents. (*Maller, 2008*)
   d. Public Gardens / Park Incivilities / Civilities: In Wichita, Kansas, a garden coordinator estimated that graffiti was reduced by about 75% and gang activity decreased after the installation of a “planting peace” community garden. (*Shefte, 2014*)

Urban Design / Land Use

There were 202 entries in the urban design category. Of 30 cells (Table 9), 8 had strong evidence of co-benefits, 5 had good evidence, and 6 had moderate evidence. In the urban design category, 4 cells had moderate or good evidence of negative effects, and 1 cell had strong evidence of negative effects. Specific findings include:

- Mixed use, greenery, street scale design, and connectivity had evidence of 4 to 5 co-benefits.
- All urban design features had strong evidence of green/environmental benefits, except evidence was good for streetscale design.
- All urban design features had evidence of economic benefits, and the evidence was strong for mixed use.
- Only greenery had strong evidence of mental health benefits. None had evidence of injury prevention benefits.
- Residential density had the most complex pattern with good evidence of negative health effects, strong evidence of environmental sustainability, and good evidence of economic benefits.

In general, we found very strong evidence of multiple health, environmental, and economic benefits of most of the urban design features. Creating walkable communities (dense, mixed use, connected streets), with substantial greenery, and pedestrian-friendly street designs can help meet multiple goals of city decision-makers. Finding ways to reduce apparent negative health effects of high density remains a challenge to city planners and politicians.

Table 9: Urban Design / Land Use Summary Scores						
**Built Environment Attribute**	**Physical Health**	**Mental Health**	**Social Benefits**	**Environmental Sustainability**	**Safety / Injury Prevention**	**Economic Benefits**
**Residential density**	19+<br>21.5(0)<br>7.5-		13.5+<br>14.5(0)	88+<br>21(0)<br>3.5-	4.5(0)<br>7.5-	15+<br>3.5(0)
**Mixed land use**	28+<br>17(0)<br>4-	4.5+<br>4-	33+<br>11(0)	95+<br>21(0)	4.5(0)<br>11-	22.5+<br>3.5(0)<br>4-
**Streetscale pedestrian design**	7.5+		7.5+	7.5+		7+
**Greenery**	20.5+<br>3.5(0)	26.5+	12+	39.5+		12+
**Accessibility & Street connectivity**	30+<br>12(0)<br>7.5-		14.5+<br>3.5(0)	35.5+<br>3.5(0)	4.5(0)	12.5+<br>3.5(0)

Highlights of Key Urban Design and Land Use Findings

1. **Physical Health**
   a. Urban greenness: Patients in hospital rooms facing a park had a 10% faster recovery and needed 50% less strong pain medication compared to patients whose rooms faced a building wall.(*Bolund, 1999*)
   b. Walkability: A 5% increase in walkability was associated with a … 0.23 point reduction in BMI. (*Frank, et al, 2006*)
   c. Density / Block size: Contrary to expectations, the hypothesised most walkable neighbourhood (high density, small block stratum) had the greatest mean and median BMI. After adjusting for demographic covariates, physical activity and clustering due to neighbourhood, no conclusive effect of population density by block size on BMI was found (β=−1.024, 95% CI −2.408 to 0.359). (*McDonald KN, Oakes JM, Forsyth A, 2012*)

2. **Mental Health**
   a. Urban greenness: People who lived in proximity to natural space had significantly improved mental health up to three years after their move. Compared to pre-move mental health scores, individuals who moved to greener areas (n = 594) had significantly better mental health in all three post-move years (P = .015; P = .016; P = .008), supporting a "shifting baseline" hypothesis. Individuals who moved to less green areas (n = 470) showed significantly worse mental health in the year preceding the move (P = .031) but returned to baseline in the post-move years. (*Alcock, et al, 2013*)
   b. Urban greenness: A moderately significant association between city forest and park and a 87% recovery rate from stress. (*Hansmann, Hug, & Seeland, 2007*)
   c. Urban greenness: Individuals reported less mental distress and higher life satisfaction when they were living in greener areas. (*White, 2013*)

3. **Social Benefits**
   a. Mixed use: Cross sectional study of 100 Seattle census tracts found businesses located in residential areas had an increased risk of burglary. (*Wilcox, et al, 2004*)

b. Density / Mixed use: Every 1 percent increase in the proportion of individuals driving to work in a neighborhood is associated with a 73 percent decrease in the odds of any individual having a neighborhood social tie and a 71 percent decrease in any individual having more ties (*Freeman, 2001*)
c. Density / Mixed use/ Connectivity: Reported crime is 5 times higher in New Urbanist layouts; 6 of the first 7 reasons burglars stated for selecting a particular property were related to access routes. (*Stone Jr., Bachman, 2000*)

4. **Environmental Benefits**
   a. Mixed use / Density: Exhaustive review. "When viewed in total, the evidence on land use and driving shows that compact development will reduce the need to drive between 20 and 40 percent…So, as a rule of thumb, it is realistic to assume a 30 percent cut in VMT with compact development. Making reasonable assumptions…[this could] reduce total transportation-related CO2 emissions …by 7 to 10 percent in 2050." (*Ewing, et al, 2008*)
   b. Walkability: Survey of 6,994 adults in Washington state. A 5% increase in walkability was associated with a 6.5% fewer vehicle miles traveled, 5.6% fewer grams of oxides of nitrogen emitted, and 5.5% fewer grams of volatile organic compounds emitted. (*Frank, et al, 2006*)
   c. Urban greenness: In Chicago, increasing tree cover in the city by 10% may reduce the total energy for heating and cooling by 5 to 10%. (*Sorensen, et al, 1997*)

5. **Economics**
   a. Walkability: Comprehensive review: making places better for walking can: boost footfall and trading by up to 40%; raise retail rents by 20%; In a study of Hong Kong included in this review, there was a 17% increase in retail rents from pedestrianisation. (*Lawlor, 2013*)
   b. Walkability: Retail properties with a Walk Score® ranking of 80 were valued 54% higher than properties with a Walk Score® ranking of 20. This was accompanied by an increase in net operating income (NOI) of 42% for the more walkable properties. Office properties showed identical higher premium values, although rental apartments showed only a 6% premium if they were in walkable locations. Walkability was associated with higher value for all types of properties. Properties with a Walk Score of 80 were worth 29% to 49% more than properties with a score of 20. Consistent with their higher values, study found higher net operating incomes for all property types as well. (*Pivo & Fisher, 2010*)
   c. Walkability: A careful study in 15 cities, controlling for a variety of contextual factors, found that shifting from average to above-average Walk Score® ratings raised the housing values by $4,000 to $34,000, depending upon the metropolitan area. The amounts tended to be greater in dense urban areas such as Chicago and San Francisco and lower in low-density cities such as Tucson and Fresno. (*Cortright, 2009*)
   d. Mixed use / Density: 60 percent of respondents favor a neighborhood with a mix of houses, stores, and other businesses that are within walking distance, rather than neighborhoods requiring driving between home, work, and recreation. Respondents indicated that while the size of a home or yard does matter, most are willing to compromise size for a preferred neighborhood and less commuting. (*Community Preference Survey, 2013*)

6. **Injury Prevention**: n/a

Transportation Systems

There were 81 entries in the transportation systems category. Of 48 cells (Table 10), 5 had strong evidence of co-benefits, 2 had good evidence, and 6 had moderate evidence. One cell had good evidence of negative impact. Specific findings include:

- Pedestrian and bicycle facilities had the best evidence of multiple co-benefits, followed by lower traffic speed and volume.

- Strong evidence of co-benefits was most evident in the injury prevention and economic domains.
- Traffic calming had strong evidence of injury prevention benefits.
- Public transport had strong evidence of economic benefits and mixed evidence of environmental benefits.
- Many cells had inadequate evidence. Even in well-studied topics, there was little study of health consequences of transportation decisions.

Environmental strategies to promote active transportation, provide public transportation, and protect pedestrians and bicyclists from automobile traffic had good to strong evidence of multiple benefits, particularly in the areas of economics, injury prevention, and environmental protection. Physical and mental health and social benefit consequences of transportation systems are poorly studied.

Table 10: Transportation Systems Summary Scores

Built Environment Attribute	Physical Health	Mental Health	Social Benefits	Environmental Sustainability	Safety / Injury Prevention	Economic Benefits
**Pedestrian / Bicycle facilities**		3+	7+	10.5+<br>3.5(0)	27.5+<br>4(0)	22.5+<br>3.5(0)
**Crosswalk markings**					6(0)<br>4-	
**Traffic calming**	3.5+	3.5(0)	3+	3+<br>3-	23+	3+
**Public Transportation**	3.5-			28.5+<br>17.5(0)		20+<br>4-
**Traffic speed/ Volume**	3.5+		3+	14+	7+	7+
**Safe routes to school**			3+	3.5+	9.5+<br>4(0)	
**Ciclovia / Play streets**			7+			3.5+
**Managed parking**				10.5+		

Highlights of Key Transportation Findings

1. **Physical Health**
   a. Traffic volume: The size of the traffic density effect is about 0.27 BMI attained units over the 10–90th percentile of the 150 m traffic distribution. This translates into about a 5% increase in attained BMI at age 18. (*Jerrett, 2010*)
   b. Traffic calming: A rise in physical component summary (PCS) scores between the first and second surveys that constituted a statistically significant improvement in physical health status. (*Morrison, 2004*)
   c. Public transportation: Adults living in blocks with TransMilenio (bus rapid transit) stations were less likely to have higher scores of health-related quality of life ($\beta = -2.2$; $P = .041$). Likewise, adults living further than 500 m from TransMilenio stations were more likely to report perceiving their health status as good/excellent but this association was marginally significant. (*Sarminento, 2011*)

2. **Mental Health**
    a. Traffic calming: Mental component summary scores (MCS) fell slightly in men and improved slightly in women between the first and second surveys, but neither change was statistically significant. (*Morrison, 2004*)

3. **Social Benefits**
    a. Ciclova: 89% of participants said the event changed their feelings for the city in a positive way and 74% felt the city was more vibrant during the event. The majority strongly agreed/agreed (88.1%) that Open Streets strengthens the local community. (*Hipp, 2013*)
    b. Public transportation: Among those who say their community is served by public transit, 75% say they are satisfied with its quality. Among residents who are denied access to mass transit because their area does not offer it, half (51%) say they are dissatisfied with the lack of availability. (*Urban Land Institute, 2013*)
    c. Traffic calming: One study found that traffic calming helps make public streets lively and friendly, encourages community interaction, and attracts customers to commercial areas. (*Litman*, 1999)

4. **Environmental Benefits**
    a. Public transportation: Public transportation produces 95% less carbon monoxide (CO), 90% less in volatile organic compounds (VOCs), and about half as much carbon dioxide (CO2) and nitrogen oxide (NOx), per passenger mile, as private vehicles. (*Shapiro, 2002*)
    b. Ped/bike infrastructure (sidewalks): The addition of sidewalks to all roadways would lead to a VMT reduction of 1.142 miles per person-day. This equates to a total of 182.938 million miles reduced across the entire population of Dane County during year 2002. Multiplying this VMT reduction amount by the average unit cost of $0.045 per vehicle-mile for motor vehicle air pollution (VTPI, 2006) gave an annual air pollution cost saving of $8.23 million. (*Guo, 2010*)

5. **Injury Prevention**
    a. Traffic calming / Ped/bike infrastructure: Modification of the built environment can substantially reduce the risk of pedestrian-vehicle crashes. Highly effective countermeasures include: single-lane roundabouts, sidewalks, exclusive pedestrian signal phasing, pedestrian refuge islands and increased intensity of roadway lighting. (*Retting, 2003*)
    b. Safe Routes to School: After SRTS interventions, annual rate of pedestrian injury decreased 33% (95% confidence interval [CI]: 30 to 36) among school-aged children (5- to 19-year-olds) and 14% (95% CI: 12 to 16) in other age groups. Annual rate of school-aged pedestrian injury during school-travel hours decreased 44% (95% CI: 17 to 65) from 8.0 injuries per 10,000 population in the pre-intervention period (2001-2008) to 4.4 injuries per 10,000 population in the post-intervention period (2009-2010) in census tracts with SRTS interventions. The rate remained virtually unchanged in census tracts without SRTS interventions (0% [95% CI: -8 to 8]). (*Dimaggio, 2013*)
    c. Traffic calming (Road Diet): Road diets typically reduce crash rates by 47% on major highways through small urban areas, by 19% on corridors in larger city suburban areas, and 29% overall. (*USDOT*)
    d. Ped/bike infrastructure (complete streets) / Traffic speeds: Lower traffic speeds as a result of complete streets tend to reduce traffic collision rates and severity, and therefore crash costs, particularly injury risk for pedestrians and cyclists. (*USDOT*)
    e. Ped/bike infrastructure (Bike lanes): There are consistent data (two review studies) showing that marked bike lanes on roads reduce motor vehicle–bicycle collisions by as much as 50%. (*Pollack, 2012*)

6. **Economic Benefits**
    a. Health care costs; Economic performance of cities: By 2040, investments in bike facilities in the range of $138 to $605 million will result in health care cost savings of $388 to $594 million, fuel savings of $143 to $218 million, and savings in value of statistical lives of $7 to $12 billion. The benefit-cost ratios for health care and fuel savings are between 3.8 and 1.2 to 1, and an order of magnitude larger when value of statistical lives is used. (*Gotschi, 2011*)

b. Traffic volume: A further externality of private motorized transport is traffic congestion that imposes significant costs on economic efficiency as time lost due to congestion reduces productivity. Congestion costs in Canada are as high as US$4.5 billion nationally of which 80% is accounted for by the country's three largest urban regions: Greater Toronto (43%), Montreal (21%) and Vancouver (17%). In the US, congestion has led urban Americans to travel 5.5 billion hours more and to purchase an extra 11 billion litres of fuel for congestion-related costs of US$121 billion in 2011.150 In 2005, the cost of congestion in Australia's eight capital cities was US$7.1 billion, comprised of private time costs (37%), business time costs (38%), extra vehicle operating costs (13%) and extra air pollution (12%). The immense economic impact of traffic congestion is further illustrated by the case of Cairo, which costs Egypt as much as 4% of its GDP. (*UNHSP*)
c. Ped/bike infrastructure (bike lanes): A conservative estimate of the annual economic impact of the bicyclists is $60 million. The annual economic impact of cyclists is almost nine times as much as the one-time expenditure of public funds used to construct special bicycle facilities in the region (Outer Banks, NC). (*Lawrie, 2004*)
d. Ped/bike infrastructure: Almost all of the studies (n=20) identified (UK and beyond) report economic benefits of walking and bicycling interventions which are highly significant, and these average 13:1. For UK interventions only the average figure is higher, at 19:1. (*Davis, 2010*)
e. Public transportation: It is estimated that every $10 million in capital investment in public transportation yields $30 million in increased business sales, and that every $10 million in operating investment in public transportation yields $32 million in increased business sales. For every $10 million invested, over $15 million is saved in transportation costs to both highway and transit users. These costs include operating costs, fuel costs, and congestion costs. (*Cambridge Systematics, 1999*)
f. Ped/bike infrastructure (bike trail): For every quarter mile nearer to an off-street bicycle trail, the median home value increases by $510 in Minneapolis-St. Paul, MN. (*Krizek, 2006*).
g. Pedestrian/bicycle infrastructure (Biking vs auto use): In Portland, OR, people who traveled to a shopping area by bike spent 24% more per month than those who traveled by car ($75.66 vs. $61.03 per month, respectively). (*Clifton, 2012*)

Schools

There were 27 entries in the school setting category. Of 18 cells (Table 11), 2 cells had strong evidence of co-benefits, 1 cell had good evidence, and 5 cells had moderate evidence. Specific findings include:

- Siting schools near the homes of students had strong evidence of environmental sustainability and moderate evidence of mental health and economic benefits.
- Having recreation facilities at schools had strong evidence of mental health and good evidence of physical health benefits.
- Shared use agreements had moderate evidence of social benefits, injury prevention, and economic benefits.

The co-benefits of school environment features were poorly studied for most outcomes.

Table 11: Schools Summary Scores

Built Environment Attribute	Physical Health	Mental Health	Social Benefits	Environmental Sustainability	Safety / Injury Prevention	Economic Benefits
**School siting**	3.5+	4.5+		21.5+	3-	4+
**Recreation facilities**	16+<br>3.5(0)	16.5+	3.5+			3.5+
**Shared use agreements**			7.5+		4+	7.5+

Highlights of Key School Findings

1. **Physical Health**
    a. Recreation facilities: Obesity was negatively related to child-reported but not parent-reported access to physical activity facilities and availability of bicycle and walking trails. (*Dunton, 2008*)
    b. Recreation facilities: The number of locked schoolyards was positively related to obesity in children BUT proximity to playgrounds, parks and play areas were unrelated to BMI in children. (*Dunton, 2008*)
    c. Recreation facilities: Weight of youth is inversely to the spatial accessibility to recreational physical activity facilities. A literature review showed 4 of 9 studies where excess weight was inversely associated to the accessibility of various recreational infrastructures. (*Casey, 2014*)
    d. Recreation facilities: Gymnasium and playground provision in 5th grade were not predictive of a lower obesity or overweight trajectory both overall and for stratifications separately by gender, obesity, or overweight status in kindergarten, household poverty, region, and climate zone. Gymnasium availability was associated with an additional 8.3 min overall and at least an additional 25 min of PE per week for schools in humid climate zones; but, no significant findings were obtained for gymnasium and playground adequacy in relation to obesity trajectory. (*Fernandes, 2010*)
    e. Recreation facilities: Schools represented 44% of potential neighborhood sites for physical activity and the number of locked schools was associated with significantly higher BMI. (*Scott, 2007*)

2. **Mental Health**
    a. Recreation facilities: Controlling for the racial/ethnic composition of a school and the percent of students receiving reduced or free lunches, schools that had a renovated schoolyard had more of their students passed the state mandated math test (*Lopez, 2008*).
    b. Recreation facilities: CRF (college recreation facility) usage, while simultaneously controlling for other important academic, financial and social fit factors, had a positive association with academic success (*Huesman, 2007*). Freshmen who used the SRC (student recreation complex) persisted (didn't drop out) at a greater rate after 1 semester and after 1 year than their counterparts who did not use the SRC. Users earned slightly higher GPAs and more credit hours at the end of the first year. This finding was of particular interest because the study also revealed that the SRC nonusers entered college with higher high school GPAs and higher ACT/SAT scores than SRC users. (*Belch, 2000*)
    c. Recreation facilities: Teachers reported that children were more social, creative, and resilient when unstructured play materials were provided on the playground. Children who were creative, rather than very physically capable, became leaders in activity; the unstructured materials promoted play between children who had not played together previously--including children who had formerly been excluded. (*Bundy, 2008*)

3. **Social Benefits**
    a. Joint use agreements: All 68 faculty/staff respondents agreed that the joint use program had a positive impact on the school and that it was beneficial to students. They reported that the program provided needed opportunities for PA, incurred social benefits (e.g., making new friends), kept students out of trouble, and promoted healthy lifestyles. (*Maddock, 2008*)
    b. Joint use agreements: Public schools can serve as community "hubs," and offer a range of medical, social, and other support services. (*Filardo, 2010*)
    c. Joint use agreements: On-site services that promote intergenerational use, such as health care, job training, or a senior center, give seniors and childless professionals a vested interest in their neighborhood school. (*Chung, 2002*)

4. **Environmental Benefits**
    a. School siting: Neighborhood schools produce less emissions than schools located on outskirts of town. Compared to a sample from existing schools, neighborhood schools would reduce traffic, produce a 13 % increase in walking and biking, and lead to a reduction of at least 15% in emissions of concern. (*Ewing, 2003*)

b. School siting: Emissions are 3–8 times lower for the neighborhood-only scenario as for the current scenario; distance traveled by automobile is more than cut in half. Busing rate drops by more than half and busing distance declines by more than an order of magnitude. (*Marshall, 2010*)
c. School siting: A Safe Route to School program in Marin County, California calculated that, after the fourth year, the percentage of children arriving and leaving school in a family car dropped from 55 percent to 42 percent, or 4,250 fewer car trips per day. This represents an annual reduction of roughly 1,000 tons of carbon dioxide emissions and 70 tons of other environmental pollutants. (*Staunton, 2003*)

5. **Economics**
   a. School Siting: The presence of a local school supports higher property values. (*Kuhlman, 2010*)
   b. School siting (community-centered schools): Keeping an existing school open increases home values in surrounding neighborhoods and helps stabilize the area and its business activity, while closing them slows the rise of home values. (*Kuhlman, 2010)*
   c. School Siting: Building new schools costs more than renovating old schools. (*Kuhlam, 2010*)
   d. School siting: Community-centered schools save on construction and operating costs. (*Kuhlman, 2010*)
   e. School siting: School construction on undeveloped sites generates many new expenses for infrastructure and government services, which eventually raise taxes for business and property owners. (*Michigan Land Use Institute*)
   f. Joint use agreements: Joint use partnerships can help the partners save money by reducing land costs and allowing partners to share costs of space and programming. Partners may bring funding for capital expenses or operating costs. (*Cooper, 2008*)
   g. Joint use agreements: Using the public school as the location for community health centers, swimming pools, libraries, or other public amenities or services, can thereby reduce overall public land assets, capital funds, and total operating costs required *(Filardo, 2010*).
   h. Joint use agreements: Joint use agreements allow a community to offer programs, facilities, and services that it might otherwise not be able to afford. (*Nathan, 2001*)

6. **Injury Prevention**
   a. Joint use agreements: Sharing community facilities such as libraries, parks and meeting spaces is a sound community strategy that not only saves money and space, but also unites the public school and neighborhood through shared uses. Restoring the public character of public schools by serving as a community hub encourages members of the community to put their own resources into the school, including volunteer maintenance, mentoring, donations and cultural resources. Moreover, as a community hub, there are more eyes on the school grounds, and thus a greater degree of safety in the school and in the neighborhood. (*Chung, 2002*)

Buildings / Workplaces

There were 39 entries in the workplace/building category (Table 12). Of 36 cells, 3 cells had good evidence of co-benefits and 3 cells had strong evidence. Specific findings include:

- Building site design features (mainly outdoor) had strong evidence of physical and good evidence of mental health benefits.
- Features of the building design had strong evidence physical health and good evidence of environmental sustainability and economic benefits.
- Physical activity programs and policies had strong evidence of economic benefits.
- 5 cells had evidence of moderate evidence.

For workplace and building features, the best evidence was for physical health and economic benefits.

Table 12: Workplaces / Buildings Summary Scores

Built Environment Attribute	Physical Health	Mental Health	Social Benefits	Environmental Sustainability	Safety / Injury Prevention	Economic Benefits
**Building siting**	4+					
**Mixed land use around worksite**				4+		4+
**Building site design**	16+	11.5+				3.5+
**Building design**	19.5+	3.5+<br>4-		12.5+		12+
**Worksite physical activity policies and programs**	8.5+	3.5+		4+		25+
**Workplace furniture design**	7+<br>3.5(0)					3.5+<br>3.5(0)

Highlights of Key Workplaces and Buildings Findings

1. **Economics: Productivity/Job Performance/Healthcare Costs**
   a. <u>Worksite health program</u>: Although the results of worksite health promotion (WHP) programs are limited as to the impact on health and long-term outcome measures, but seems positive. Specific risk factors account for 25% of total employer health care costs, particularly employees with multiple co-morbidities of modifiable risk factors; ones with 7 of the risk factors (tobacco use, hypertension, hypercholesterolemia, overweight/obese, high blood glucose, high stress and lack of PA) costs employees 228% more in health care costs compared with those lacking any of these risk factors. Reviews found that because WHP programs vary a lot it is hard to declare the cost of savings but at least $3 savings per $1 invested. (*Goetzel & Ozminkowski, 2008*)
   b. <u>Worksite health program</u>: Employees who participate in WHP programs have lower absentee rates, where the more comprehensive the program is, the greater the improvement (ex: 12%). (*Aldana & Pronk, 2001; Bertera, 1993*)
   c. <u>Worksite health program</u>: Large employers adopting wellness programs see substantial positive returns, even within the first few years after adoption. Employee wellness programs costs $144 and generates $358 worth of savings per person through reducing healthcare costs. Medical costs fall about $3.27 for every dollar spent on wellness pro- grams, and absentee day costs fall by about $2.73 for every dollar spent. (*Baicker et al., 2010*)
   d. <u>Worksite fitness center</u>: Participation in worksite fitness centers is significantly associated with improved productivity and reduced work absence. Additionally those who did not join the fitness center were nearly twice as likely as participants to report health-related work limitations in time management and physical work, and 1.5 times as likely to report limitations in overall output. Assuming an average wage of US$200 per day, it was calculated that nonparticipants cost $258 more in lost work time per employee than participants. (*Burton et al., 2005*)
   e. <u>Building design</u>: The “StairWELL to better Health” CDC intervention is a low-cost intervention (less than $16,000) that increases stair users (involves painting and carpeting, framed artwork, motivational signs, and music to the stairwell). Infrared beams were used to track the number of stair users. (*StairWELL CDC*)
   f. <u>Building design</u>: Lighting, particularly daylighting (lighting through daylight) has consistently shown to be important. School children in well lit with daylight received test scores 15% higher than those in classrooms with little or no daylighting. Additionally, daylighting design is linked to a 15% reduction in

absenteeism in office environments, and increases between 2.8-20% due to luminance levels have been found in other studies. Due to individual variation and preferences, providing the means of thermal and ventilation control to individuals is ideal. Personal control over temperature, air supply, sound masking and lighting are associated with a 3% increase in productivity. (*CABE, 2004*)

g. Building design: In a case study in CA, Thayer identified 50% savings in lighting, cooling and ventilation energy as well as a 15% reduction in absenteeism, due to an integrated daylighting design that harmonized layout, orientation, and windows. (*CABE, 2004*)

2. **Mental Health**
   a. Building design: High-rise housing is associated with elevated psychological distress, especially among low-income mothers. The quality of the neighborhood, in terms of social and physical attributes, are associated with greater psychological distress and poorer cognitive development in children in lower quality neighborhoods. (*Evans, 2003*)

3. **Physical Health (Chronic Diseases)**
   a. Worksite health program: Comprehensive worksite health promotion programs, that include 1) training and support of coordinators; 2) health risk appraisals; 3) on-site classes, safety meetings, and self-help options; 4) environmental changes, e.g., smoking policy, cafeteria offerings, and blood pressure machines; and 5) recognition, decreased the number of employees with three or more risk factors by 14% and risk levels improved among high-risk individuals in 6 of the 7 risk factors (i.e. BP, cholesterol, etc.) over two years. (*Bertera, 1993*)
   b. Standing desk: A sit-stand workstation paradigm reduces musculoskeletal complaints without considerably affecting data entry efficiency. (*Huseman et al., 2009*)
   c. Standing desk: Differences as high as 25% have been attributed to differences in employees who report being comfortable vs. uncomfortable. Even improved workstations with ergonomic chairs are associated with a 23% increase in computer data entry and editing. (*CABE, 2004*)
   d. Building design: Air quality studies have shown that buildings that improve delivery of outside air are attributed to a decrease in Sick Building Syndrome (SBS). SBS is related to productivity, and it is estimated that a 10% reduction in reported SBS symptoms is associated with a 1.1% in productivity among employees. (*CABE, 2004*)

4. **Environmental Benefits**
   a. Mixed use: Residents of mixed-use neighborhoods use less motorized travel than those in purely residential areas, where the average distance per trip was half of those in single use areas; also residents used alternative transportation more--12.2% of the time compared to 3.9% of the trips in single use areas. This can reduce VMT by at least 4% for an entire region or, in specific dense sections, VMT can be reduced by as much as 17.6%. (*Ewing & Kreutzer, 2006*)

Review of Grey Literature in Europe

Cavill Associates, based in London, were commissioned to search the grey (not peer reviewed) literature for studies of co-benefits in the European region. The purpose was to ensure incorporation of international literature, and we expected most such literature to be from Europe. They found 31 reports with relevant information, mostly from advocacy organizations, and they found 59 mentions of co-benefits of activity-friendly environments. Their examples of specific and quantitative findings are included here. Their findings are also included in the matrix tables reported above.

Co-benefits Identified in European Studies

1. **Economic Benefits**, including positive economic impact; improved tourism; increased housing prices; job creation; community cohesion.
   a. Proximity to parks: Moderate to strong evidence that urban parks have a positive impact on the value of nearby property (houses, apartments, land), although it is important to keep the limitations of the hedonic pricing methods – applied in the large majority of the studies - in mind (*Konijnendijk C, et al,*

2013)

b. Ped/bike infrastructure (bicycle): Annual economic benefit of cycling in the EU -27 of at least 205bn Euros. The largest single benefit is health (80% of the total). The economic impact of cycling for tourism and the cycling industry amounts to 62 bn Euros; Congestion easing through cycling saved 24.2bn Euro (in 2010); fuel savings were 2.7-5.9 bn; reduced CO2 emissions 1.4-3.0bn Euros; reduced air pollution 0.9bn Euro; reduced noise pollution 0.3bn Euro (*Davies N, et al, 2012*)
c. Walkability: Walkability affects consumer transport costs. Improved walkability allows consumers to save on vehicle expenses ("Affordability," VTPI 2008). For example, one study found that households in automobile-dependent communities devote 50% more to transportation (more than $8,500 annually) than households in communities with more accessible land use and more multi-modal transportation systems (less than $5,500 annually) (*Litman, 2011*)

2. **Environmental Benefits,** including general environmental benefits; improved air quality; reduced noise; reduced energy use and Carbon dioxide emissions; Integration of environmental and health concerns; improved biodiversity, air quality, water management; greater use of green space.
   a. Cycling as transport: Between 1990 and 2007 greenhouse gas emissions from transport increased by 36% in the EU. By 2050the EU has set a target to reduce green house gas emissions by 80-95% (60% will need to come from transport sector). Emissions from bicycles are 10 times less than those from motorized vehicles. If levels of cycling in EU were equivalent to those of Denmark, bike use would help achieve 12-26% of the 2050 target dependant on which transport mode the bike replaces. (*Blondel, et al, 2011*)
   b. Spatial Planning: There are 4 key spatial planning issues identified as likely to have more significant impacts on health; transportation public spaces, housing and flooding.1/4 of the global disease burden is attributable to environmental factors. . The report found strong evidence that: the provision of space increases exercise; reductions in traffic reduces air pollution; green space improves mental health; moderate exercise improves health outcomes; provision of space to exercise needs to be safe and easily accessed. (Boyce and Pate, 2009)
   c. Transport policy: Transport uses 30 per cent of total energy. Since the end of the 1990s, overall energy consumption has been increasing across Europe. (*Grenier and Cavill, 2007*)

3. **Injury Prevention,** including reductions in injuries due to increase in modal share of walking and cycling and decline of motorised transport volume and speed.
   a. Road design: Road design has a key influence on speed, excess and inappropriate speed contributes to around 30% of fatal crashes in high income countries. Vulnerable road users are at particular risk. Pedestrians have a 90% chance of surviving a car crash at speeds below 30 kph. There is increased risk of death or serious injury between the ages of 10 - 11 years, with boys at greater risk than girls (65% of those killed or seriously injured are boys. 74% of casualties are on minor roads and the likelihood of dying as a car occupant is 5.5 times higher if parents are unemployed. Recommendations: Senior public health official required to support local highways authorities; Highways authorities should introduce engineering measures to reduce speeds in streets that are primarily residential or where cycle and walking levels are high. (*NICE, 2010*)
   b. Cycling City Policy: The number of cyclists killed or injured in accidents involving more than one party was reduced by 20 per cent between 1999-2002. (*Troelson et al, no date*)
   c. Transport policy: From the mid 1990s to 2000, road traffic fatalities declined in several EECA and SEE countries, probably due to the economic recession (since lower growth is correlated with lower transport volumes) rather than road safety strategies. From 2000 to 2004-5 SEE countries showed a slight decrease in the number of persons killed in traffic. (*Grenier & Cavill, 2007*)

4. **Mental Health Benefits,** including improvements to stress, wellbeing, mental health and cognitive function.
   a. Green environments: Compared with exercising indoors, exercising in natural environments was associated with greater feelings of revitalization and positive engagement, decreases in tension, confusion, anger, and depression, and increased energy. However, the results suggested that feelings of calmness may be decreased following outdoor exercise. Participants reported greater enjoyment and

satisfaction with outdoor activity and declared a greater intent to repeat the activity at a later date. (*Thomson et al, 2011*)

   b. Green space: Green spaces associated with greater wellbeing and mental health and cognitive function. "Strong body of evidence which suggests that physical activity in green spaces has stronger mental health benefits than physical activity in non-green spaces, and that "more passive forms of usage" can also have a beneficial impact on mental well-being and cognitive function." (*BoP consulting, 2013*)
   c. Green Space: There is strong evidence that access to green spaces improves mental health, although the evidence that it increases physical activity is less strong (1). Use of green spaces is associated with: a decrease in health complaints, improved blood pressure and cholesterol levels, reduced stress, improved general health perceptions and a greater ability to face problems (2). Sixty-three per cent of people in social grade AB (managerial and professionals) said they visited green spaces on a weekly basis, compared with 47 per cent in grade DE (manual workers, shop workers, apprentices, casual labourers, state pensioners and the unemployed). (*King's Fund, 2013*)

5. **Social Benefits,** including social interaction; opportunities for children's play; social cohesion; reduced fear of crime; livability; accessibility.
   a. Walkable environments: Increased walking is associated with increased social interaction, reduced crime and fear of crime, increased perceptions of safety, and the development of social capital — the network of interactions between individuals and their communities. In addition, walking brings economic benefits to both urban and rural areas. Areas with higher walking levels have been shown to have higher property prices. (*De Moor, 2013*)
   b. Walkable neighbourhoods. Poor quality neighbourhoods can have a negative impact on the health of those who live in them. It is important that housing and streetscape design are considered as part of a neighbourhood that go towards building social relationships as positive contributions toward health. Mixed communities with a range of housing types and tenures, well-designed walkways, cycle routes and streets are most likely to reduce health inequalities and can help to avoid segregation within a community. Neighbourhoods with nearby parks and green space help to support opportunities for physical activity. Such areas are particularly helpful to children and their carers and older people by reducing the risk of social isolation. Good design of these spaces encourages greater community ownership of the environment and reduces negative effects such as vandalism and the under-use of facilities. (*Royal Town Planning Institute, 2009*)
   c. Green Space: Enhanced cognitive and motor skills and socialisation for children via spaces for play and challenge. Greater social interaction and community cohesion through inclusive, free space. "Children playing in green spaces displayed higher levels of creative play, played for longer, and more collaboratively than children playing in built-up spaces. These findings are supported by a 2000 Norwegian study, which found that playing in woodland provided a more stimulating and varied play environment for children, and noticeably improved their motor fitness". (*BoP Consulting, 2013*)

Special Report on Active Living and Climate Change

Some of the most consequential co-benefits of activity-friendly environments are likely to be related to climate change. For example, active transportation can substitute for automobile trips and reduce carbon emissions. More parks and street trees can improve carbon sequestration. The relevant literature is scattered across many disciplines, so we commissioned Ding Ding and Klaus Gebel, based in Australia, to conduct a review of scientific and grey literature on the climate change co-benefits. In their extensive report, they include summaries of findings and identify promising case studies. Their findings have been incorporated into the matrix tables above.

Co-benefits Identified in Climate Change Literature

1. **Urban Planning**
   a. Density: Examined determinants of urban travel greenhouse gas emissions based on a travel survey in the Quebec City Region of Canada. They found that compared to residents in the city center (the highest residential density), those leaving in old suburbs, new suburbs, and the periphery (the lowest residential

density and farthest from the city) produced 19%, 27%, and 70% more emissions from traveling. Specifically, they also found that if one moves to a neighborhood that is 10% more dense, his/her emission would reduce by 1.2%. (*Barla, et al, 2011)*

b. Walkable neighborhoods: Estimated CO_2 equivalent emissions based on the 2006 Household Activity Survey in the Puget Sound Region of Washington State. The key findings were that households located in denser and well-mixed neighborhoods produce a smaller amount of emissions. In addition, intersection density has an inverse association with emissions. Residential density, entropy (land use mix), and intersection density were inversely associated with CO_2 emissions in both urban and suburban areas. A 100% increase in all three built environment factors could lead to 31.2-34.4% reductions in transportation emissions. (*Hong and Goodchild, 2014*)

2. Transportation

a. Transport and land use policies: The South-western city of Freiburg is considered the environmental capital of Germany. Over the last few decades, a number of transport and land-use policies have encouraged more walking, cycling and public transport use. For instance, an extensive network of bike paths and lanes was created, the city center was converted into a pedestrian only zone, new light rail lines were built and existing ones extended, a land use plan was developed that centers new developments around public transport stops, Germany's first transferable flat-rate monthly public transport ticket was introduced, and thousands of bike parking spots were created in the city. Through these and other measures over the last three decades in Freiburg the number of bicycle trips tripled, public transport ridership doubled, and the share of trips by automobile declined from 38% to 32%. Since 1990, motorization rates have leveled-off and per-capita CO_2 emissions from transport have fallen. Many of the strategies mentioned here would be transferable to car-oriented cities around the world. (*Buehler & Pucher, 2011)*

b. Public transportation (BRT): Estimated household CO_2 emissions in 23 diverse neighborhoods in Jinan, China, based on household survey data collected in 2009-2010. They found that residents living near BRT (bus rapid transit) had 13% lower CO_2 emissions compared to those who did not live near BRT. The difference in CO_2 emissions was likely due to residents' preference to use BRT when opportunities present. In Jinan, BRT offers comfortable and convenient transit conditions, and it is overall energy efficient. (*Guo, et al, 2013*)

3. Parks and Green Space

a. Greenery: It has been well understood that plants can reduce CO_2 concentration in the atmosphere through carbon sequestration, a process of "removing carbon from the atmosphere and depositing it in a reservoir". Trees play a particularly important role in capturing and storing atmospheric CO_2 in vegetation, soils, and biomass products. Resent estimates suggest that trees and forests in U.S. urban areas store 643 million tons of carbon, and the total annual gross carbon sequestration was estimated to be 25.6 million tons. (*Nowak, et al, 2013*)

b. Greenery: In 1995, in order to promote multiple environmental and health benefits, Taiwan's government started to establish air quality enhancement zones (AQEZs). By 2010, a total of 504 AQEZs had been established, including greening landfill restoration sites, bare-land sites, waste disposal sites, green open spaces, metropolitan bike paths, and metropolitan parks. The main approach of the greening process was tree planting. In 2010, the investigators sampled and surveyed 9761 trees from 48 AQEZs and found that the total forest carbon storage in the 48 sites was 296.73 tons and the annual sequestration was 157.64 tons of CO_2 per year. Given that most trees were still young, carbon sequestration by the trees will increase over time. AQEZs provide significant and direct benefits to urban areas. (*Wang & Lin, 2012*)

Conclusions

When the results from all features are combined (Table 13), there is impressive evidence of co-benefits in all physical activity settings. When settings are designed to be activity-friendly, there is good reason to expect not only more physical activity, but also other health, environmental, and economic benefits. This table represents 418 findings.

Of the 30 cells in the matrix, 22 have strong evidence and two had good evidence of co-benefits. Five cells had inadequate evidence, and only 1 cell had evidence of a net negative effect. Specific findings include:

- Each setting had strong evidence of at least 3 of the 6 co-benefits, and parks and trails had strong evidence of all 6 co-benefits. Thus, for each setting there are multiple features that can be designed to both facilitate physical activity and produce co-benefits.
- Of particular relevance to mayors and other government leaders responsible for balancing budgets, activity-friendly environments had strong evidence of economic benefits. A broad range of economic benefits was documented, such as increased home value, greater retail activity, reduced health care costs, and improved productivity.
- Activity-friendly design in all settings had strong evidence of environmental co-benefits based on reduced pollution and carbon emissions.
- There were many gaps in evidence of co-benefits in the schools and workplace settings.
- Another major gap was evidence of the health consequences of environments that support active travel.
- There was little evidence of negative consequences of activity-friendly environments. However, in the urban design setting there was some evidence of negative physical health and injury outcomes, mainly related to high residential density. However, the overall pattern of results indicated overwhelmingly positive effects for numerous important outcomes from activity-friendly environment designs.

Table 13: Quantitative Estimates of Co-Benefits by Setting

Built Environment Attribute	Physical Health	Mental Health	Social Benefits	Environmental Sustainability	Safety / Injury Prevention	Economic Benefits
**Open spaces / Parks / Trails**	57.5+<br>3.5(0)	93+	42.5+<br>4(0)	20+<br>4(0)	23+	19+<br>4(0)
**Urban design / Land use**	105+<br>54(0)<br>19-	31+<br>4-	80.5+<br>29(0)	265.5+<br>45.5(0)<br>3.5-	13.5(0)<br>18.5-	69+<br>10.5(0)<br>4-
**Transportation systems**	7+<br>3.5-	3+<br>3.5(0)	23+	70+<br>21(0)<br>3-	67+<br>14(0)<br>4-	56+<br>3.5(0)<br>4-
**Schools**	19.5+<br>3.5(0)	21+	11+	21.5+	4+<br>3-	15+
**Workplaces / Buildings**	55+<br>3.5(0)	18.5+<br>4-		20.5+		48+<br>3.5(0)

We used the same criteria for level of evidence for this summary table as we did in the setting-specific tables. Though it was "easier" to generate positive (dark green) findings in the summary table, we believe the approach is justified. Within each setting there are several ways (features) to create activity-friendliness. If there is good evidence that one of those features is related to the co-benefit, then that strategy can be used to improve that setting. If several features with weaker evidence can be applied to the design of a setting, it is reasonable to conclude this constitutes strong evidence for the overall design of the setting. The fact that the 3/4 of the cells in the summary table had strong evidence simply

indicates there is good evidence of multiple co-benefits in all settings, and several features can be used to achieve the co-benefits.

International Evidence on Co-Benefits of Physical Activity-Friendly Environments

Because coders noted the country of origin for specific studies and reports, it is possible to summarize the evidence by country (Table 14). This is important because physical inactivity is a global problem, and it is useful to know the extent to which the findings about co-benefits are similar across countries. Though we were not able to conduct separate analyses by country, we can summarize the extent to which the evidence is international. The table tallies the number of findings by country and for each grade of evidence.

Most of the international evidence came from review papers (evidence grade of 4.5 and 4), for which it was not possible to identify all the specific countries. The mix of non-US and US studies varies by review paper, and it was not possible to determine the international contribution. Most of the country-specific findings were in specific studies (evidence grade of 3.5).

Table 14: Number of Findings, by Country and Grade of Evidence

	NUMBER OF FINDINGS, BY EVIDENCE GRADE						
**COUNTRY**	**4.5**	**4**	**3.5**	**3**	**2**	**1**	**Total**
International	26	40	11	3	11	1	**92**
Australia			8	2			**10**
Canada		1	12				**13**
China			3				**3**
Columbia			7				**7**
Denmark			3	2			**5**
Germany			8				**8**
Hong Kong			1				**1**
Ireland			2				**2**
Netherlands			4	4			**8**
New Zealand			1				**1**
Norway			6				**6**
Spain			2				**2**
Switzerland			2				**2**
Taiwan			1				**1**
Turkey			1				**1**
United Kingdom	3	5	10	1	12	1	**32**
United States	7	35	81	12	37		**172**
**Total**	**36**	**81**	**163**	**24**	**60**	**2**	**366**

Almost half the studies (47%) were from US-based studies, and 25% of the findings were from international reviews. Thus, about 28% were from non-US studies representing 16 countries. Only the United Kingdom, Canada, and Australia contribution more than 10 findings to the review, but 5 or more findings were from Columbia, Denmark, Germany, the Netherlands, and Norway. Not considering the international reviews, the percent of findings from specific countries other than the US was 35% for parks/trails/open space (8 countries), 33% for urban design/land use (10 countries), 23% for transportation (8 countries), 6% for schools (2 countries), and 22% for buildings/workplaces (3 countries).

Though worldwide coverage was less than desired, and international findings were dominated by the English-speaking world, half of the findings included international studies. Our inability to search in languages other than English certainly reduced our ability to identify international literature.

Public Support for Physical Activity-Friendly Environments and Related Policies

Public opinion is an important input into the decision-making of public officials in a democracy, in part because officials are elected to represent the will of their constituents. Public opinion polls are a common political tool, and there is evidence from surveys in the US that many people want more and better physical activity-friendly environments. In this section we summarize a few polls and studies of people's preferences.

Two large national surveys in the US (about 6000 and 12,000 adults) assessed support for walkable communities in their areas and assessed trends from 2003 to 2005. In 2003 there was substantial publicity about studies showing people living in suburbs were less active and more likely to be obese and have hypertension than those living in urban, walkable areas. In 2003, 44% expressed support for building more walkable communities where they lived. This support increased significantly to 59% in 2005. Support was strong among all groups except rural residents and increased among all groups. Public support for developing walkable communities is strong, widespread, and growing. Walkable communities had appeal because they were seen as child- and elderly-friendly places (Handy, et al, 2008).

A recent random sample survey of 1200 adults in the US was conducted by the Urban Land Institute, an organization of real estate developers. They found more than half of Americans prefer neighborhoods that are close to shops, have a mix of incomes, and have public transportation. Neighborhoods that are close to a mix of shops, restaurants, and offices are especially appealing to African Americans (75%), members of generation Y (62%), single people (60%), renters (60%), and college graduates (60%). Over 60% of Americans planning to move in the next five years would prefer to settle in mixed-use communities. Many want communities that have access to public transportation (52%) (Urban Land Institute, 2013).

Though the surveys above demonstrate strong support for walkable mixed use communities with access to public transit, it appears that people are not able to find homes in such communities. A study of over 3000 people in Atlanta examined preferences for walkable communities and assessed the types of neighborhoods where people actually live. Many people were “mismatched” and did not live in their preferred neighborhood type. The biggest mismatch was for people who wanted to live in walkable neighborhoods but did not. This suggests walkable neighborhoods are under-supplied (Frank, et al, 2007). An important reason for the under-supply is that zoning laws in most cities favor or require the separate-use suburban-style development that reduces the ability of the real estate industry to respond to the market's demand for walkable communities.

Because policies, including funding priorities, determine what kinds of built environments are constructed, it is useful to assess opinions about relevant policies. About 1800 randomly sampled adults in the US rated their support of various policies that could increase physical activity. Between 89% and 92% of both women and men stated a preference for more local government funds to be devoted to walking/jogging trails, recreation centers, and bike paths. There was also strong support (86% to 88%) for zoning laws to require walking and biking paths (Brownson, et al, 2001).

Bicycling is the least used mode of transportation in the US, and it is well documented that the largest barrier to bicycle use is concern about safety from cars. A study of about 1800 adults assessed current bicycle use and projected changes if bicycling was made safer from traffic. If bicycling was safer, bicycle riding at least once per week might increase from 8% to 40% of adults. Forty-four percent of people who did not own a bicycle said they would start riding at least once per week. The projected increase in bicycling was greatest among racial and ethnic minority participants, populations who tend to be at highest risk of chronic diseases (Sallis, et al, 2013). This study suggests that many people would support changes in transportation policies that invest more in protecting bicyclists from traffic.

In summary, there is good evidence that most US adults want more walkable neighborhoods, like to live in mixed use neighborhoods, and want safer places to ride bicycles. There is strong support for more government funding for walking and bicycling facilities and recreation centers. The evidence we found says that US adults are in favor of the kinds of activity-friendly environments that help people be active and produce important co-benefits.

Disparities in Access to Physical-Activity Friendly Environments

One of the biggest problems with health in the US is that there are disturbingly large disparities in life expectancy and health outcomes when comparing high- and low-income people and when comparing those from majority and minority racial-ethnic groups. There are many reasons for these persistent disparities, but increasing focus is being placed on the role of environments. It is known that exposure to toxic chemicals tends to be greater in low-income areas, but evidence is also accumulating that low-income neighborhoods in the US provide lower quantity and quality of opportunities for physical activity.

Active Living Research compiled the research on this topic. There were three main conclusions of this review (Taylor & Lou, 2011).

- Residents of lower-income neighborhoods generally lacked well-maintained sidewalks, attractive scenery, and trees, all features that make for safer and more enjoyable physical activity.
- Residents of lower-income neighborhoods were less likely to have parks in their neighborhood, and those without nearby recreation facilities tended to be less active.
- Residents of lower-income neighborhoods usually had more crime in their area as well as more physical and social disorder such as graffiti, litter, drug use, and public drinking.

As an example of a specific study, about 2200 adults from two regions of the US reported on environmental characteristics of their neighborhoods. Their neighborhoods were selected to vary widely on both income and walkability. Participant perceptions of specific neighborhood characteristics were compared across lower- and higher-income groups. Residents of lower-income neighborhoods reported (Sallis, Slymen, et al, 2011):

- Fewer walking and cycling features, such as well maintained sidewalks, pedestrian paths, and bike trails;
- Fewer aesthetically pleasing features, such as landscaping, trees that provide shade, interesting sights, and attractive buildings;
- Fewer pedestrian and traffic safety features, such as safe crosswalks and slow traffic speeds;
- Less safety from crime, including comfort of walking at night, and neighborhood crime rates;
- Fewer nearby recreation areas such as, basketball courts, soccer fields, tennis courts;
- Fewer gyms and fitness facilities within walking distance.

Unfortunately there is growing evidence that low-income people in the US live in neighborhoods that have fewer public and private recreation facilities, less-safe environments for walking and bicycling, less-safe intersections, and less attractive scenery that might encourage them to be active. Lower-quality physical activity environments for low-income people and communities of color is a social injustice that likely contributes to higher rates of chronic diseases and higher health care costs. Targeting investments in physical activity environments in low-income neighborhoods could bring a particularly good return on investment.

Countries vary greatly on income and health inequalities, so findings about disparities in the US will not be applicable to many other countries.

Strengths and Limitations of the Review

The primary strength of the literature review was the breadth of topics explored. For each setting we identified several features that were related to physical activity, and for each feature we evaluated for 6 types of co-benefits. We searched the international scientific literature and the grey literature using numerous search terms to account for differences in terminology across fields. We solicited recommendations from experts in all the settings. We coded the level of evidence of each source and created summary scores of the evidence by weighting each finding by the rating of the source. To avoid basing our findings on lower quality evidence, such as poorly-substantiated claims in advocacy documents, we did not include lower levels of evidence in our summaries. However, all relevant documents found in the search are coded in the evidence tables. To be even more conservative, summary scores represented in the final matrix tables are based on positive findings minus null and negative findings.

The main limitations of the review were a consequence of the breadth. Because of the large number of topics searched for, it was not possible to conduct a systematic review or a quantitative meta-analysis. A requirement of systematic reviews is assessment of the quality of each study, but this was not feasible. We relied on existing reviews whenever

possible. When reviews were available, we did not include the individual studies, but we did attempt to find more recent studies. A different coder conducted each search, and though they received some training and supervision, they were not experts in literature reviews or syntheses. There are undoubtedly differences across topics in thoroughness of search, classification of levels of evidence, and identification of findings to highlight.

Though we searched the international literature, we were only able to do so in English. This limited our ability to identify and include the full range of international studies. Nevertheless one-quarter of the findings were from specific non-US countries, and another quarter of findings were from reviews that included international literature. The majority of the international literature was from English-speaking countries. We were unable to compare results from US and non-US countries.

Other limitations include publication bias that favors positive findings and inclusion of grey literature that may be more biased.

We expect there will be some objections to our summary scores. Though we do not encourage the score to be interpreted literally as the actual strength of evidence, we believe the scores do provide a rough indication of the extent of evidence--pro, con, and neutral. There are certainly limitations since reviews that receive a score of 4.5 may summarize 10 studies or 100 studies. However, if the net scores for level of evidence high, there is reason to have high confidence in the finding for a connection between a feature and an outcome. Please consider the summary scores rough indicators, not absolute or true measures.

Recommendations

The most important conclusion of this review is that creating communities, transportation systems, schools, and buildings that make physical activity attractive and convenient also produces a wide range of other benefits for communities. Benefits were found for green/environment, economic, and multiple dimensions of health outcomes. It is often said that a good solution solves multiple problems, so building places that are good for physical activity should be considered a superlative solution.

Rates of chronic disease are high in countries at all income levels, and these rates are increasing fastest in low and middle income countries (UN, 2011). Health care costs are unsustainable and climbing in most countries. The mental health disorder of depression creates the highest burden of disease worldwide. Injuries are the biggest cause of death among youth people worldwide. Every country and city is looking for ways to improve economic growth. Pollution from automobiles is a major threat to health in most countries and is making many cities unlivable. The consequences of climate change are expected to be the worst human-made disasters in history. It seems inconceivable that making cities better for physical activity can contribute to solving all of these problems. However, more parks and trails, walkable community designs, more sidewalks and bike paths, better public transit, and schools and workplaces within walking and biking distance of students and workers could improve the functioning of cities in many ways.

We urge mayors, other city officials, and staff in multiple departments to consult these findings as an aid in decision-making. There are many reasons to make a city better for physical activity, so it is reasonable to consider the full range of effects.

One of the more surprising findings was that all five physical activity settings could be designed so they have positive effects on economic outcomes. Based on the specific studies we found, many groups could enjoy economic benefits of activity-friendly environments, including reduced government spending on infrastructure, home owners, real estate developers, health insurance companies, employers, retailers, commercial property owners, and taxpayers. This is an extremely broad coalition, and we suspect most of them are not aware of the economic benefits of activity-friendly environments.

There is no one environmental feature that will solve the problem of physical inactivity. Some features support active transportation and others support active recreation. An active living city supports both types of physical activity. An international study clearly showed that the more activity-friendly features in a neighborhood, the more active people are (Sallis, 2009). Thus, it is the pattern of multiple features that seems to be important. Several features need to work together. The combination of walkable urban designs and transportation environments that support walking and biking

is needed to facilitate active transportation. If people can walk or bike safely to well-designed parks, they may get twice the physical activity. Children's physical activity is likely to be maximized when they can walk or bike safely to nearby schools and stay after school to use high-quality recreation facilities. Physical activity of workers is likely to optimized when the workplaces are in walkable communities served well by public transit, the building has attractive and accessible stairs, and there are facilities for showers and exercise.

There are multiple features of each setting that can be designed well to yield the multiple co-benefits (Table 15). Rather than thinking that designing one feature of a transportation system or school is sufficient, we encourage decision-makers and designers to consider how all features of a setting can be optimized for physical activity and multiple other benefits. Explicit and conscious design of the best possible physical activity-promoting settings can be expected to pay off with multiple benefits, so additional costs are likely to be excellent investments.

Table 15: Best Evidence of Environmental Features with Strong Multiple Benefits
(at least "moderate" evidence of three benefits)

BUILT ENVIRONEMNT ATTRIBUTE	EVIDENCE
**Open Spaces/Parks/Trails**	
Park presence/proximity	3 strong, 2 good
Programs, promotion, and events	4 moderate
**Urban Design/Land Use**	
Mixed land use	3 strong, 1 moderate (1 strong negative)
Greenery	3 strong, 2 good
Streetscale pedestrian design	4 moderate
Accessibility and connectivity	1 strong, 2 good 1 moderate (1 good evidence of negative)
**Transportation**	
Pedestrian/bicycle infrastructure	2 strong, 2 moderate
Reduced traffic speed and volume	1 strong, 2 moderate
**Schools**	
School siting	1 strong, 2 moderate
Shared use agreements	3 moderate
**Buildings/Workplaces**	
Building design	1 strong, 2 good
Physical activity policies and programs	1 strong, 2 good

Changing policies about built environments is usually a politically difficult process, opposed by vested interests and vocal citizens. Evidence of the benefits of change may not be sufficient to overcome voices of negativity. Fortunately, we can expect many powerful voices to speak in favor of creating activity-friendly environments. Real estate developers can make more profits. Homeowners will likely see their properties appreciate. Retailers can expect more business. Most importantly, the majority of people, at least in the US, support more walkable communities, more walking and bicycling facilities, and more and better parks and recreation facilities. People want to live in walkable communities but cannot because zoning laws in the US favor single-use, automobile-dependent developments. Though there is opposition to every proposal, support for activity-friendly environments appears to be widespread and growing.

The studies showing that people with low incomes have less activity-friendly and less-safe neighborhoods should stimulate action. Low-income status is one of the biggest drivers of poor health, and neighborhood design and physical activity amenities may be contributing to the poor health. Thus, targeting improvements to low-income communities could help reduce health disparities and may produce additional co-benefits in economic development. However,

targeted improvements to low-income neighborhoods should be planned so the current residents benefit and are not priced out as the neighborhood is enhanced.

The evidence in this report should reduce resistance to making multiple environmental changes to promote physical activity. Creating more activity-friendly features produces additional co-benefits, including economic ones, which will be experienced by a broad range of stakeholders. Thus, a reasonable conclusion based on the evidence is that the more we invest in helping residents be active, the better physical/mental/social health, environmental health, and economic health of the city. People want more activity-friendly neighborhoods, so policy changes to create them are likely to be popular. It would make sense for city leaders to compete with other cities in creating the best activity-friendly environments.

References

1. Alkhajah, TA, Reeves, MM, Eakin, EG, Winkler, EAH, Owen, N, & Healy, GN. (2012). Sit-Stand Workstations: A Pilot Intervention to Reduce Office Sitting Time. *American Journal of Preventive Medicine*, 43(3), 298-303.

2. Badland, H & Schofield, G. (2005). Transport, Urban Design, and Physical Activity: An Evidence-based Update. *Transportation Research Part D: Transport and Environment*, 10(3), 177-196.

3. Bauman, AE, Reis, RS, Sallis, JF, Wells, JC, Loos, RJF, & Martin, BW. (2012). Correlates of Physical Activity: Why Are Some People Physically Active and Others Not? *The Lancet*, 380(9838), 258-271.

4. Brownson, RC, Baker, EA, Housemann, RA, Brennan, LK, & Bacak, SJ. (2001). Environmental and Policy Determinants of Physical Activity in the United States. *American Journal of Public Health*, 91(12), 1995-2003.

5. Crespo, NC, Sallis, JF, Conway, TL, Saelens, BE, & Frank, LD. (2011). Worksite Physical Activity Policies and Environments in Relation to Employee Physical Activity. *American Journal of Health Promotion*, 25(4), 264-271.

6. Davison, KK & Lawson, CT. (2006). Do Attributes in the Physical Environment Influence Children's Physical Activity? A Review of the Literature. *International Journal of Behavioral Nutrition and Physical Activity*, 3, 19.

7. Ding, D, Sallis, JF, Kerr, JK, Lee, S, & Rosenberg, DE. (2011). Neighborhood Environment and Physical Activity among Youth: A Review. *American Journal of Preventive Medicine*, 41(4), 442-455.

8. Frank, LD, Saelens, BE, Powell, KE, & Chapman, JE. (2007). Stepping Toward Causation: Do Built Environments or Neighborhood and Travel Preferences Explain Physical Activity, Driving, and Obesity? *Social Science & Medicine,* 65(9), 1898-1914.

9. Godbey, G & Mowen, A. (2010). *The Benefits of Physical Activity Provided by Park and Recreation Services: The Scientific Evidence*. Ashburn, VA: National Recreation and Park Association. Available at: http://www.nrpa.org/uploadedFiles/nrpa.org/Publications_and_Research/Research/Papers/Godbey-Mowen-Research-Paper.pdf.

10. Handy, S, Sallis, JF, Weber, D, Maibach, E, & Hollander, M. (2008). Is Support for Traditionally Designed Communities Growing? Evidence from Two National Surveys. *Journal of the American Planning Association*, 74, 209-221.

11. Heath, GW, Brownson, RC, Kruger, J, Miles, R, Powell, KE, Ramsey, LT, et al. (2006). The Effectiveness of Urban Design and Land Use and Transport Policies and Practices to Increase Physical Activity: A Systematic Review. *Journal of Physical Activity and Health*, 3(Suppl 1), S55-S76.

12. Lafleur, M, Gonzalez, E, Schwarte, L, Banthia, R, Kuo, T, Verderber, J, et al. (2013). Increasing Physical Activity in Under-Resourced Communities through School-based, Joint-Use Agreements, Los Angeles County, 2010-2012. *Preventing Chronic Disease: Public Health Research, Practice, and Policy*, 10.

13. Lee, IM, Shiroma, EJ, Lobelo, F, Puska, P, Blair, SN, & Katzmarzyk, PT. (2012). Effect of Physical Inactivity on Major Non-communicable Diseases Worldwide: An Analysis of Burden of Disease and Life Expectancy. *The Lancet*, 380(9838), 219-229.

14. McMillan, TE. (2009). *Walking and Biking to School, Physical Activity, and Health Outcomes. A Research Brief.* Princeton, NJ: Active Living Research, a National Program of the Robert Wood Johnson Foundation. Available at: http://activelivingresearch.org/walking-and-biking-school-physical-activity-and-health-outcomes.

15. Miles, R. (2008). Neighborhood Disorder, Perceived Safety, and Readiness to Encourage Use of Local Playgrounds. *American Journal of Preventive Medicine*, 34(4), 275-281.

16. Molnar, BE, Gortmaker, SL, Bull, FC, & Buka, SL. (2004). Unsafe to Play? Neighborhood Disorder and Lack of Safety Predict Reduced Physical Activity among Urban Children and Adolescents. *American Journal of Health Promotion*, 18(5), 378-386.

17. Ng, SW & Popkin, BM. (2012). Time Use and Physical Activity: A Shift Away from Movement Across the Globe. *Obesity Reviews*, 13(8), 659-680.

18. Nicoll, G & Zimring, C. (2009). Effect of Innovative Building Design on Physical Activity. *Journal of Public Health Policy*, 30(Suppl 1), S111-123.

19. Proper, KI, Koning, M, van der Beek, AJ, Hildebrandt, VH, Bosscher, RJ, & van Mechelen, W. (2003). The Effectiveness of Worksite Physical Activity Programs on Physical Activity, Physical Fitness, and Health. *Clinical Journal of Sport Medicine*, 13(2), 106-117.

20. Rothman, L, Buliung, R, Macarthur, C, To, T, & Howard, A. (2014). Walking and Child Pedestrian Injury: A Systematic Review of Built Environment Correlates of Safe Walking. *Injury Prevention*, 20, 41-49.

21. Sallis, JF, Bowles, HR, Bauman, A, Ainsworth, BE, Bull, FC, Craig, CL, et al. (2009). Neighborhood Environments and Physical Activity among Adults in 11 Countries. *American Journal of Preventive Medicine*, 36(6), 484-490.

22. Sallis, JF, Adams, MA, & Ding, D. (2011). Physical Activity and the Built Environment. In J Cawley (Ed.), *The Oxford Handbook of the Social Science of Obesity* (pp. 433-451). New York: Oxford University Press.

23. Sallis, J., Slymen, D., Conway, T., Frank, L., Saelens, B., Cain, K., & Chapman, J. (2011). Income Disparities in Perceived Neighborhood Built and Social Environment Attributes. *Health & Place,* 17(6), 1274-1283.

24. Sallis, JF, Floyd, MF, Rodriguez, DA, & Saelens. (2012). Role of Built Environments in Physical Activity, Obesity, and Cardiovascular Disease. *Circulation*, 125, 729-737.

25. Sallis, JF, Conway, TL, Dillon, LI, Frank, LD, Adams, MA, Cain, KL, et al. (2013). Environmental and Demographic Correlates of Bicycling. *Preventive Medicine*, 57(5), 456-460.

26. Sarmiento, OL, Torres, A, Jacoby, E, Pratt, M, Schmid, TL, & Stierling, G. (2010). The Ciclovia-Recreativa: A Mass-Recreational Program with Public Health Potential. *Journal of Physical Activity and Health*, 7(Suppl 2), S163-S180.

27. Spengler, JO. (2012). *Promoting Physical Activity through the Shared use of School and Community Recreational Resources. A Research Brief.* Princeton, NJ: Active Living Research, a National Program of the Robert Wood Johnson Foundation. Available at: http://activelivingresearch.org/promoting-physical-activity-through-shared-use-school-and-community-recreational-resources.

28. Stewart, O, Moudon, AV, & Claybrooke, C. (2014). Multistate Evaluation of Safe Routes to School Programs. *American Journal of Health Promotion*, 28(sp3), S89-S96.

29. Taylor, W & Lou, D. (2011). *Do All Children Have Places to Be Active? Disparities in Access to Physical Activity Environments in Racial and Ethnic Minority and Lower-Income Communities. A Research Synthesis.* Princeton, NJ: Active Living Research, a National Program of the Robert Wood Johnson Foundation. Available at: http://activelivingresearch.org/do-all-children-have-places-be-active-disparities-access-physical-activity-environments-racial-and.

30. Torres, A, Sarmiento, OL, Stauber, C, & Zarama, R. (2013). The Ciclovia and Cicloruta Programs: Promising Interventions to Promote Physical Activity and Social Capital in Bogota, Colombia. *American Journal of Public Health*, 103(2), e23-e30.

31. United Nations. (2011). *Draft Political Declaration of the High-level Meeting on the Prevention and Control on Non-communicable Diseases*. Available at http://www.un.org/en/ga/ncdmeeting2011/pdf/NCD_draft_political_declaration.pdf.

32. United States Environmental Protection Agency, Development, Community, and Environment Division. (2006). *Parking Spaces / Community Spaces: Finding the Balance through Smart Growth Solutions*. Washington, DC: U.S. Environmental Protection Agency. Available at: http://www.epa.gov/smartgrowth/pdf/EPAParkingSpaces06.pdf.

33. Urban Land Institute. (2013). *America in 2013: A ULI Survey on Views on Housing, Transportation and Community*. Washington, DC: Urban Land Institute. Available at http://www.uli.org/research/centers-initiatives/terwilliger-center-for-housing/research/community-survey/.

34. Zimring, C, Joseph, A, Nicoll, GL, & Tsepas, S. (2005). Influences of Building Design and Site Design on Physical Activity: Research and Intervention Opportunities. *American Journal of Preventive Medicine*, 28(2 Suppl 2), 186-193.

ABOUT THE PROGRAM

Active Living Research, a program of the Robert Wood Johnson Foundation, stimulates and supports research to identify environmental factors and policies that influence physical activity for children and families to inform effective childhood obesity prevention strategies, particularly in low-income and racial/ethnic communities at highest risk. Active Living Research wants solid research to be part of the public debate about active living.

Active Living Research
University of California, San Diego
3900 Fifth Avenue, Suite 310
San Diego, CA 92103-3138
www.activelivingresearch.org
